# Four new species of *Capsicum* (Solanaceae) from the tropical Andes and an update on the phylogeny of the genus

**DOI:** 10.1371/journal.pone.0209792

**Published:** 2019-01-16

**Authors:** Gloria E. Barboza, Carolina Carrizo García, Segundo Leiva González, Marisel Scaldaferro, Ximena Reyes

**Affiliations:** 1 Instituto Multidisciplinario de Biología Vegetal (IMBIV), CONICET, Córdoba, Argentina; 2 Facultad de Ciencias Químicas, Universidad Nacional de Córdoba, Córdoba, Argentina; 3 Department of Botany and Biodiversity Research, University of Vienna, Vienna, Austria; 4 Museo de Historia Natural, Universidad Privada Antenor Orrego, Trujillo, Perú; 5 Facultad de Ciencias Exactas, Físicas y Naturales, Universidad Nacional de Córdoba, Córdoba, Argentina; 6 Centro de Investigaciones Fitoecogenéticas de Pairumani, Cochabamba, Bolivia; Indiana University Bloomington, UNITED STATES

## Abstract

Four new species of *Capsicum* (Capsiceae, Solanaceae) from Andean tropical forests in South America are described. *Capsicum benoistii* Hunz. ex Barboza sp. nov. (incertae sedis) is endemic to a restricted area in south-central Ecuador and is most similar to the more widespread *C*. *geminifolium* (Dammer) Hunz. (Colombia, Ecuador, and Peru). *Capsicum piuranum* Barboza & S. Leiva sp. nov. (Andean clade) is found in northern Peru (Department Piura) and is morphologically most similar to *C*. *caballeroi* M. Nee of the Bolivian yungas (Departments Santa Cruz and Cochabamba) but closely related to C. *geminifolium* and *C*. *lycianthoides* Bitter. *Capsicum longifolium* Barboza & S. Leiva sp. nov. (Andean clade) occurs from northern Peru (Departments Amazonas, Cajamarca, and Piura) to southern Ecuador (Province Zamora-Chinchipe), and is morphologically most similar to *C*. *dimorphum* (Miers) Kuntze (Colombia, Ecuador, and Peru). *Capsicum neei* Barboza & X. Reyes sp. nov. (Bolivian clade) is endemic to southeastern Bolivia (Departments Chuquisaca and Santa Cruz) in the Boliviano-Tucumano Forest, is morphologically most similar to another Bolivian endemic species *C*. *minutiflorum* Rusby (Hunz.), and is closely related to *C*. *caballeroi*. Complete descriptions, illustrations, distributions and conservation assessments of all new species are given. Chromosome numbers for *C*. *piuranum* and *C*. *longifolium* are also provided. Three of the new species were included in a new phylogenetic analysis for *Capsicum*; their positions were strongly resolved within clades previously recognized in the genus.

## Introduction

*Capsicum* L. (Capsiceae, Solanaceae) comprises ca. 35 species [1] native to tropical and temperate Central and South America, Mexico and the West Indies. The genus includes the sweet and hot chili peppers, which have been popular from ancient times and at present are of great commercial interest, not only for the taste and colour of their fruits [2], but also because of their essential oils and the presence of capsaicin [3,4]. Capsaicin is the pungent principle of hot chili peppers, which are commonly used as spices and also as medicines, mainly applied topically to relieve neuropathic pain and itching [5].

The five major cultivated and economically most important species of *Capsicum* are *C*. *annuum* L., *C*. *chinense* Jacq., *C*. *frutescens* L., now widely cultivated throughout Europe, the southern United States, Africa, India, and China, and C. *baccatum* L. and *C*. *pubescens* Ruiz & Pav., cultivated predominantly in South America [6–9].

In the phylogenetic reconstructions of Solanaceae [10, 11], *Capsicum* has been recovered along with *Lycianthes* as sister taxa and the only members of tribe Capsiceae (Solanoideae). However, a recent genomic study analyzing transcriptome sequences in members of both genera supports the paraphyly of *Lycianthes* with respect to *Capsicum*. This has important implications for the taxonomy of the Capsiceae, in that the circumscription of *Capsicum* should either expand, and/or *Lycianthes* should be split into segregate genera if monophyly serves as a foundation for taxonomy [12].

The most inclusive phylogeny of the genus, which samples the majority of the recognized *Capsicum* species and is based on three molecular markers (*matK*, *psbA-trnH* and *waxy*), has been recently published [1]. In this analysis, *Capsicum* forms a monophyletic clade with *Lycianthes* as its sister group. Eleven well-supported clades (four of them monospecific) have been recognized, named the Andean, Caatinga, Flexuosum, Bolivian, Longidentatum, Atlantic Forest, Purple Corolla, Pubescens, Tovarii, Baccatum, and Annuum clades. However, the placement of some species within the genus is not strongly resolved or their closest allies are still unclear.

Among the major regions of the Andes, the central and northern Andes are the tropical American biodiversity hotspots where it is estimated that as many as 10 to 20% of species could still remain undescribed [13]. As a complete monograph for the genus is in progress (Barboza et al. in prep.), extensive field explorations have been carried out in the tropical Andes of Ecuador, Peru, and Bolivia as well as exhaustive revisions of the collections in the herbaria to achieve a deep knowledge of the morphological variability present in many species. As a result, several new species have been identified in the Andean montane forests and are here given formal description with information on karyology and their position in the phylogeny of the genus.

## Materials and methods

### Ethics statement

The permit for collecting plant material in Ecuador was provided by Ministerio del Ambiente (MAE-Loja and MAE-Zamora-Chinchipe); in Bolivia and Peru collections were made under agreements with the Herbario Nacional de Bolivia, Universidad Mayor de San Andrés (LPB), and Museo de Historia Natural (HAO), Universidad Privada A. Orrego, Trujillo, respectively.

### Taxonomy

Several field trips were made in Bolivia and Ecuador during 2017 & 2018, and in Peru in 2017, to search for the species. Fresh material was preserved in FAA (formaldehyde–acetic acid–ethanol) or alcohol (70°) to perform measurements of reproductive organs using a Zeiss Stemi 2000-C stereomicroscope at 6.5–50 × magnification or trichomes using a Leitz light microscope at 10–40 x magnification. Descriptions were based on living plants observed during field work and examination of herbarium specimens loaned from or inspected at the following 19 herbaria: CORD, DAV, F, G, GB, GH, HAO, HSB, HUSA, HUT, LOJA, MO, NY, QCA, QCNE, S, US, USM, W [14]. Measurements of dried material were made from dissections of flowers or fruits rehydrated in hot water. Information about flower, fruit, and seed color was taken mainly from our own observations in the field; we also tested pungency of immature and mature fruits by tasting them in the field.

The geographic distribution for each species was plotted using QGIS 2.8 (QGIS Development Team, 2018) and was based on georeferenced data of all the herbarium collections analyzed. Conservation status was assessed using IUCN criteria B, geographic range in the form of B1 (EOO: extent of occurrence) and B2 (AOO; area of occupancy) [15]. The extent of occurrence and area of occupancy were calculated using the Geospatial Conservation Assessment Tool GeoCAT [16, 17].

### Nomenclature

The electronic version of this article in Portable Document Format (PDF) in a work with an ISSN or ISBN will represent a published work according to the International Code of Nomenclature for algae, fungi, and plants, and hence the new names contained in the electronic publication of a PLOS article are effectively published under that Code from the electronic edition alone, so there is no longer any need to provide printed copies.

In addition, new names contained in this work have been submitted to IPNI, from where they will be made available to the Global Names Index. The IPNI LSIDs can be resolved and the associated information viewed through any standard web browser by appending the LSID contained in this publication to the prefix http://ipni.org/. The online version of this work is archived and available from the following digital repositories: PubMed Central and LOCKSS.

### Karyology

One population each of *C*. *longifolium* (Barboza and Leiva 4821) and *C*. *piuranum* (Barboza and Leiva 4841) were studied. Somatic chromosomes were observed in squashed root meristems obtained from germinated seeds. The root apices were fixed in 3:1 ethanol: acetic acid mixture for 12 hr after a pretreatment in mM 8- hydroxyquinoline solution for two hr at room temperature and two hr at 4°C. The material was kept at -20°C until examination. Fluorochrome-stained chromosomes of somatic metaphases were observed in pectinase-cellulase-macerated root tip squashes [18]. Fluorescent chromosome banding to reveal the type and distribution of constitutive heterochromatic regions was performed using the triple staining technique (CDD) with the fluorochromes chromomycin A3, distamycin A and 4'-6-diamidino-2-phenylindole (CMA/DA/DAPI) [19]. Metaphase chromosomes were observed and photographed with epifluorescence using an Olympus BX61 microscope equipped with the appropriate filter sets (Olympus, Shinjuku-ku, Tokyio, Japan) and a JAI CV-M4 + CL monocromatic digital camera (JAI, Barrington, N.J., USA). For the karyotype description, chromosomes were arranged in groups according to the position of the centromere and in order of decreasing size within each type. Chromosome terminology followed Levan et al. [20]. The ideograms were based on chromosome measurements of fluorochrome banded metaphase plate photomicrographs, according to Moscone et al. [21]. The number of metaphases and individuals used for the karyotype analysis of each taxon is shown in S1 Table.

### Phylogeny

#### DNA extraction and sequencing

Genomic DNA was extracted, using the DNeasy Plant Mini kit (Qiagen, Valencia, USA), following the manufacturer’s instructions, from silica-gel dried leaves of *C*. *longifolium*, *C*. *piuranum*, and *C*. *neei*, as well as from other 23 *Capsicum* species, one from *Lycianthes* and one from *Dunalia* (S3 Table). Five molecular markers were sequenced: intergenic spacers *psbA-trnH*, *rpl32-trnL*, *ndhF-rpl32* and *trnL-trnF* from the chloroplast genome, and the single-copy nuclear gene *waxy* (GBSSI, granule-bound starch synthase, exons 2 to 7). With the exception of a few samples (see below), *psbA-trnH* and *waxy* were amplified as previously done for *Capsicum* species [1], *rpl32-trnL* after Sang et al. [22], *ndhF-rpl32* according to Miller et al. [23], and *trnL-trnF* following Taberlet et al. [24]. For *rpl32-trnL*, *ndhF-rpl32*, and *trnL-trnF*, the reaction mixtures were prepared using the ReddyMix PCR Master Mix (Thermo Fisher Scientific, Waltham, USA) as for *psbA-trnH* [1]. DNA extraction for some samples yielded degraded and/or insufficient concentrations of DNA. In those cases, amplifications were achieved following two different strategies: a. using the Phusion Green Hot Start II High-Fidelity PCR Master Mix (Thermo Fisher Scientific, Waltham, USA) for *waxy*, and b. after a two-steps nested reaction, the first one using the ReddyMix PCR Master Mix and the second one with the Phusion Green Hot Start II High-Fidelity PCR Master Mix, for the plastid markers. The following reaction mixture was used with the Phusion Green Hot Start II High-Fidelity PCR Master Mix in all cases: 1 μL DNA, 5 μL mix, 1 μl each primer (5 μM), 0.3 μL dimethyl sulphoxide, 0.1 μL bovine albumin, and 2.6 μL trehalose. For amplification with the Phusion Green Hot Start II High-Fidelity PCR Master Mix, the conditions were adjusted for each marker (S2 Table), following the manufacturer’s recommendations. In all cases, PCR products were cleaned using a combination of the enzymes exonuclease I (Exo I, Thermo Scientific) and thermosensitive alkaline phosphatase (FastAP, Thermo Scientific), following Werle et al. [25], and sequenced on an automated capillary sequencer [Macrogen Inc. (Seoul, South Korea) and University of Vienna (Vienna, Austria)].

#### Phylogenetic analysis

A bayesian inference (BI) analysis was conducted to explore the affinities of three of the new species proposed. A preliminary test (see next paragraph for details) was made to have a first insight on the placement of the new species within the framework of the 11 clades recognized in *Capsicum* [1]. A single sample of representative species of most clades (exception made for the Longidentatum clade) was included and only the sequences of the plastid markers were used for this scope. Based on the result (not shown), the number of samples was increased for the clades were the new species were resolved to perform the definitive analyses; cultivated species/varieties were avoided in all steps, except for *C*. *pubescens*. The materials studied and their collection data are listed in S3 Table. New sequences were obtained for most samples for the five markers, which were deposited in GenBank, and a few were downloaded from GenBank (mostly used in Carrizo García et al. [1]; S3 Table).

The DNA sequences obtained were aligned using MEGA 7 [26] and combined in a single data set. BI analysis was done in MrBayes 3.2.2 [27], using a Markov chain Monte Carlo (MCMC) search with five million generations. Excluding the first 25% of the trees discarded as burn-in, the trees obtained were used to build a majority-rule consensus tree along with posterior probability values (PP; strong support ≥ 0.95, moderate support 0.94–0.70, weak support ≤ 0.69). The GTR+R nucleotide substitution model was selected *a priori* following the Akaike Information Criteria implemented in jModelTest 2.1.3 [28].

## Results and discussion

### Taxonomic treatment

***Capsicum benoistii* Hunz. ex Barboza, sp. nov.** [urn:lsid:ipni.org:names: 77192556–1]. Type: Ecuador. Tungurahua: Baños, 3 Apr 1931 (fl), *M*. *R*. *Benoist 4204* (holotype, P).

Fig 1

**Fig 1:**
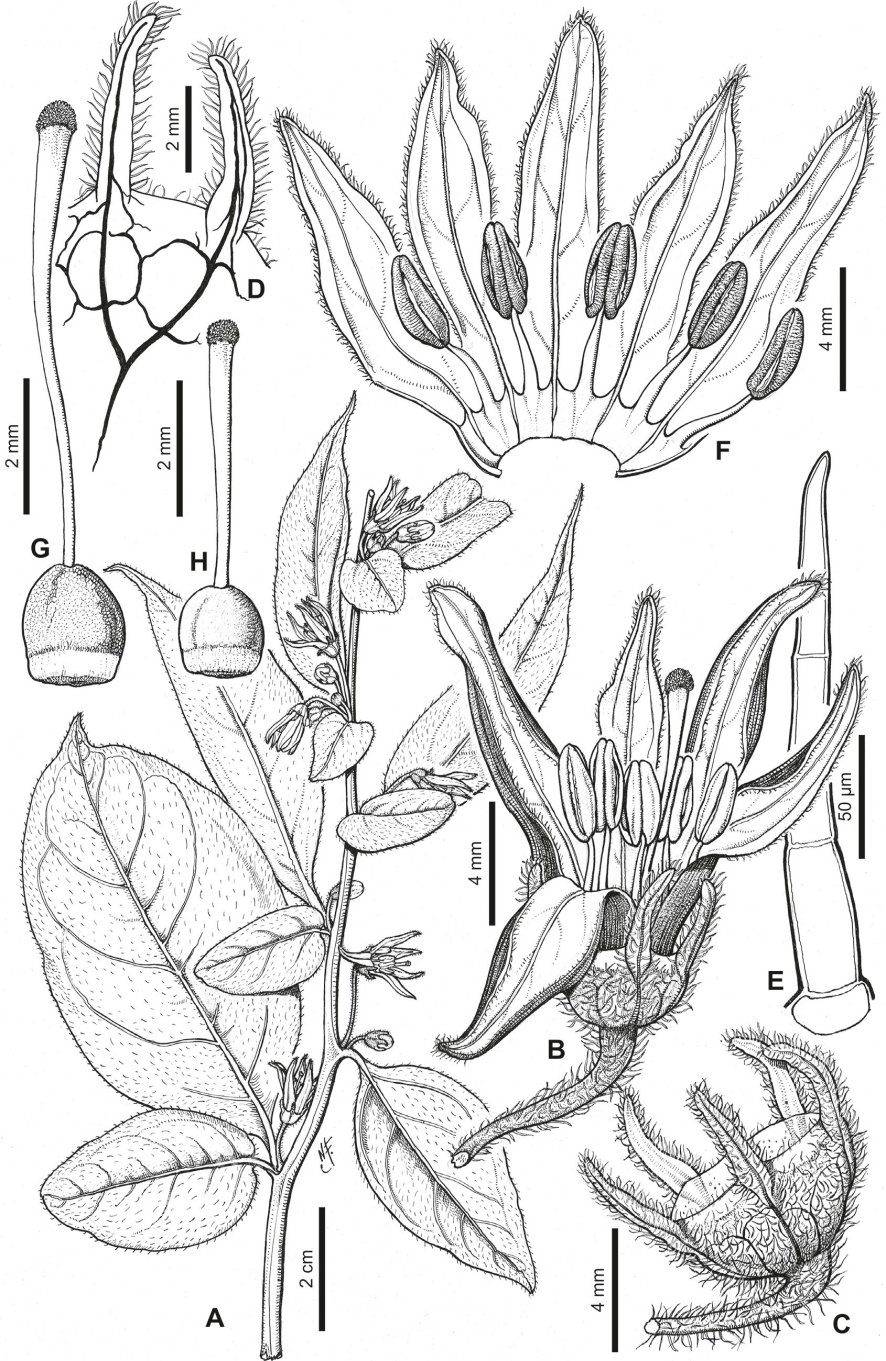
*Capsicum benoistii* Hunz. ex Barboza. (A) Flowering branch. (B) Flower. (C) Calyx. (D) Section of the calyx showing venation in tube and appendages. (E) Trichome of the calyx. (F) Opened corolla. (G, H) Gynoecium, with long and short style, respectively. Drawn by N. de Flury.

**Diagnosis**. Similar to *Capsicum geminifolium* (Dammer) Hunz. but differing in the length of the flowering pedicels, the shape of the corolla, and the presence of heterostylous flowers.

**Description**. Shrubs few branched. Young stems light brown, glabrous or pubescent, striate; bark of older stems dark brown, glabrous, striate; lenticels absent. Sympodial units difoliate, geminate, leaf pair markedly anisophyllous in size and shape. Leaves simple, membranaceous, discolorous, adaxial surface dark green, abaxial surface light green, glabrous or with simple antrorse trichomes 0.3–1.1 mm long adaxially and abaxially, trichomes more abundant on main veins; the larger leaves with blades 8.5–12 cm long, 2.8–6 cm wide, ovate or elliptic, major veins 4–5 (6) on each side of midvein, base asymmetric and attenuate, margin entire, apex long-acuminate; petioles 0.5–1.0 cm long, glabrous or glabrescent with trichomes like those of the leaves; the minor leaves 2.4–6 cm long, 1.7–4 cm wide, ovate or elliptic, major veins 3–4 on each side of midvein, base rounded, asymmetric, margin entire, apex acute or rounded; petioles 0.1–1 cm long, glabrescent or pubescent. Flowers in fascicles of 3–6; flowering pedicels filiform, striate, pendent, not geniculate at anthesis, 1.3–2 cm long, moderately to densely pubescent, the trichomes simple, non-glandular, multicellular, antrorse, 0.30–0.75 mm long. Flower buds ovoid. Calyx 2–2.5 mm long, ca. 5 mm wide, cup-shaped, thick, the margin truncate, pubescent with the same trichomes as pedicels, with 5 appendages 2.5–3.5 mm long, ca. 0.5 mm wide, thick, erect, subulate, inserted close to the margin, pubescent with the same trichomes as calyx tube. Corolla ca. 12–13 mm long, deeply stellate, thick, without interpetalar tissue; tube ca. 3 mm long, glabrous inside and outside; lobes ca. 9 mm long, ca. 2 mm wide, narrowly triangular, erect, glabrous adaxially and abaxially, the tips and margins pubescent. Stamens 5, equal, filaments equal, 3–3.2 mm long, glabrous, inserted on the corolla 1.5 mm from the base, with inconspicuous auricles at point of insertion; anthers ca. 3 mm long, not connivent, elliptic. Ovary 1.3–1.7 mm long, 1.2–1.5 mm diam, subglobose, glabrous; nectary ca. 0.3 mm long, inconspicuous; long style ca. 6.5 mm long, short style ca. 3.6 mm long, widening distally, glabrous; stigma 0.3 mm long, 0.5 mm wide, globose. Berry unknown.

**Fig 2:**
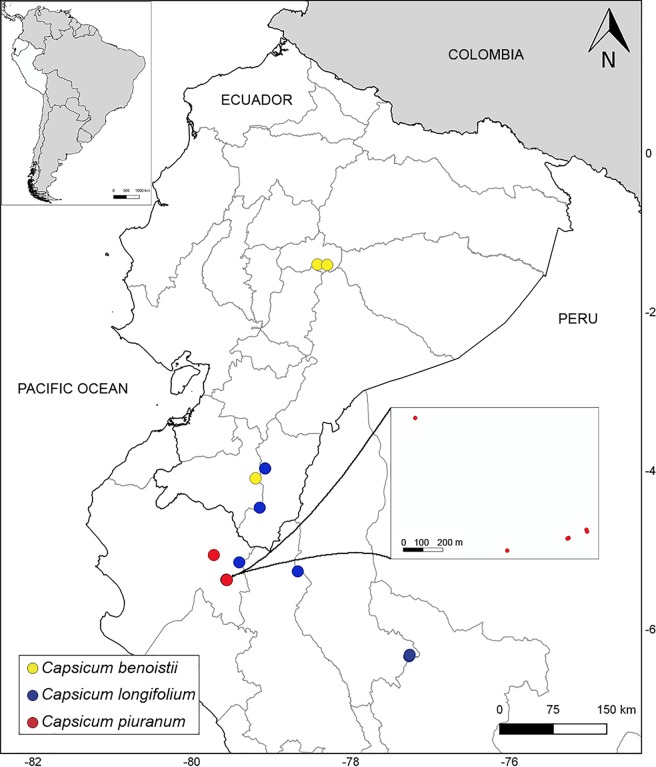
Distribution of *Capsicum* species.

**Distribution and ecology**. Endemic to a restricted area in central-southern Ecuador (Tungurahua, Loja, Fig 2) growing in thickets in montane forests, between 1500–2600 m elevation.

**Phenology**. Flowering from March to May. Fruiting time unknown.

**Etymology**—The new species is named in honor to Raymond Benoist (1881–1970), a French botanist, who collected in French Guyana, Morocco and Ecuador; the holotype is a nice specimen collected by her in 1931.

**Species Conservation Assessment**. Following the IUCN Red List Criteria (IUCN 2017), this species is proposed as Endangered (EN). The extent of occurrence is calculated to be 2050 km2 (Criterion B1 < 5000 km2, Endangered), the area of occupancy, 12 km2 (Criterion B2 < 500 km2, Endangered) and the species is known from only three localities (Criterion B1a ≤ 5, Endangered). It is possible that its geographic range has declined (EOO and AOO, Criterion B2b i & ii) because the species has not been collected since 1978 despite recent intensive searches in the same locations.

**Additional specimens examined**. **ECUADOR. Loja**: Pueblo Nuevo, 04°05'51''S, 79°11'55''W, 2580 m, 21 May 1978 (fl), *F*. *Vivar & Estudiantes 1066* (LOJA); **Tungurahua**: Río Verde Grande, 1500 m, 30 Mar 1956 (fl), *E*. *Asplund 20070* (S).

*Capsicum benoistii* was identified as a new species by the late Solanaceae specialist Armando T. Hunziker (CORD) who annotated the epithet name *benoistii* on the specimen housed at P (Benoist 4204), but this name was never published. It is a poorly known species collected only three times in Ecuador; none of these collections have fruits. Extensive recent field explorations in Tungurahua were unsuccessful in finding this species. It is distinctive in its deeply lobed stellate corolla (lobes three times longer than the tube, Fig 1B) and in the presence of heterostylous flowers (Fig 1G and 1H). These features plus the short flowering pedicels (1.3–2 cm long) distinguish *C*. *benoistii* from *C*. *geminifolium*, which has funnel-shaped corollas lobed about halfway, homostylous flowers, and longer pedicels (5 cm long).

The presence of heterostylous flowers as in *C*. *benoistii* is unusual among *Capsicum* species. It has been reported in *C*. *baccatum* L. varieties: var. *baccatum* [29] and var. *umbilicatum* (Vell.) Hunz. & Barboza [30, 31], and observed in other species (*C*. *tovarii* Eshbaugh, P.G.Sm. & Nickrent and *C*. *pubescens* Ruiz & Pav., Barboza pers. obs.). This character deserves careful field observations to ascertain if short-styled flowers produce fruits.

As some data are still unknown (e.g. corolla color, fruit and seed characters, and chromosome number) for this species and freshly collected leaf material is not available for DNA extraction, we cannot suggest in which of the different clades of the current phylogeny of *Capsicum* [1] it could be placed.

***Capsicum longifolium* Barboza & S. Leiva, sp. nov.** [urn:lsid:ipni.org:names: 77192558–1]. Type: Ecuador. Zamora-Chinchipe: Area of Estación Científica San Francisco, road Loja-Zamora, ca. 35 km from Loja, transect Q2, 03°58’S, 79°04’W, 1900 m, 12 Jun 2005 (fl, fr), *F*. *A*. *Werner 1548* (holotype, QCA [QCA-160608]; isotypes, LOJA, NY [NY-01130066]).

Figs 3 and 4

**Fig 3:**
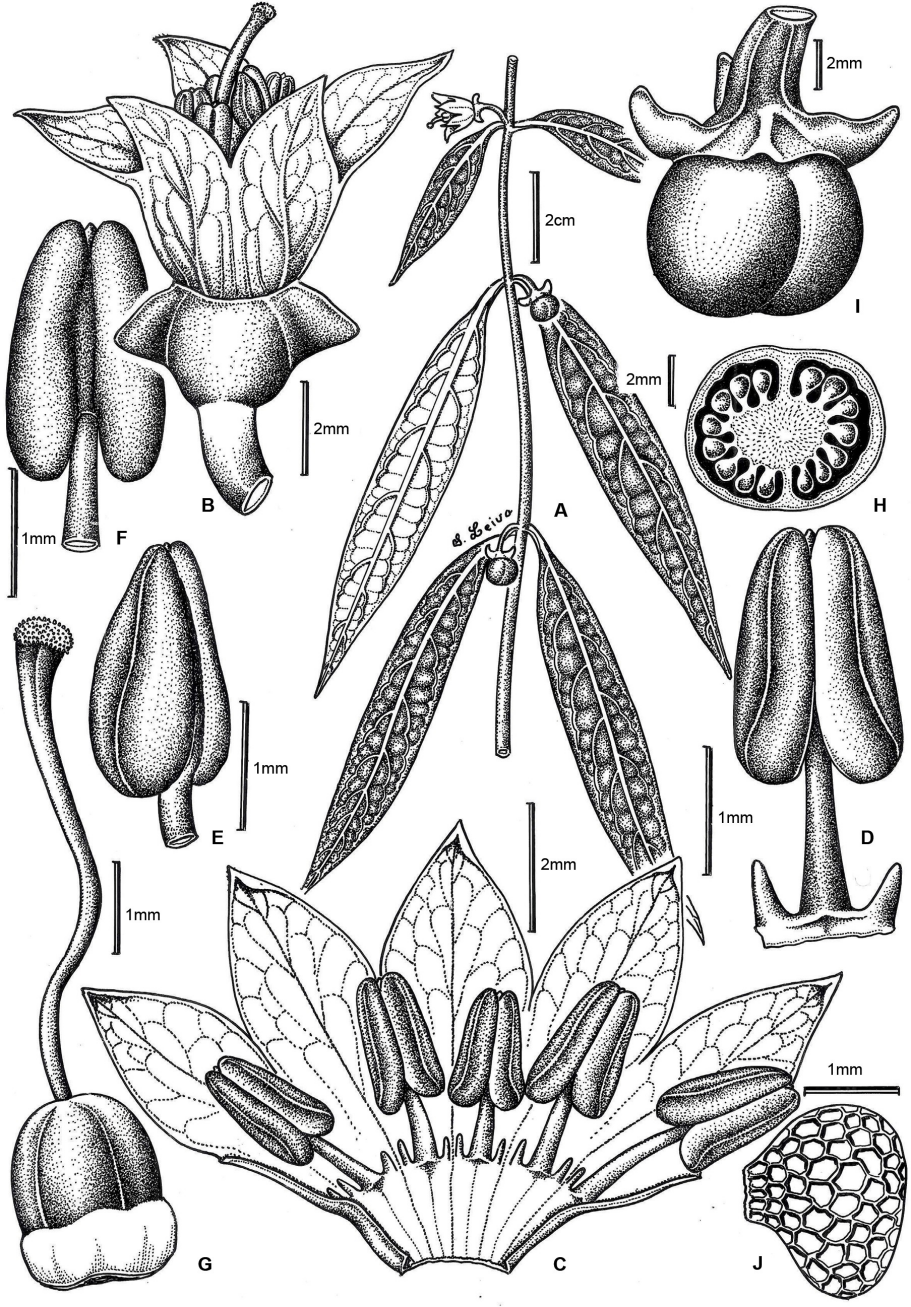
*Capsicum longifolium* Barboza & S. Leiva. (A) Flowering and fruiting branch. (B) Flower. (C) Opened corolla. (D, E, F) Anther, ventral, lateral and dorsal view, respectively. (G) Gynoecium. (H) Ovary in cross section. (I) Fruit. (J) Seed. Drawn by S. Leiva González.

**Fig 4:**
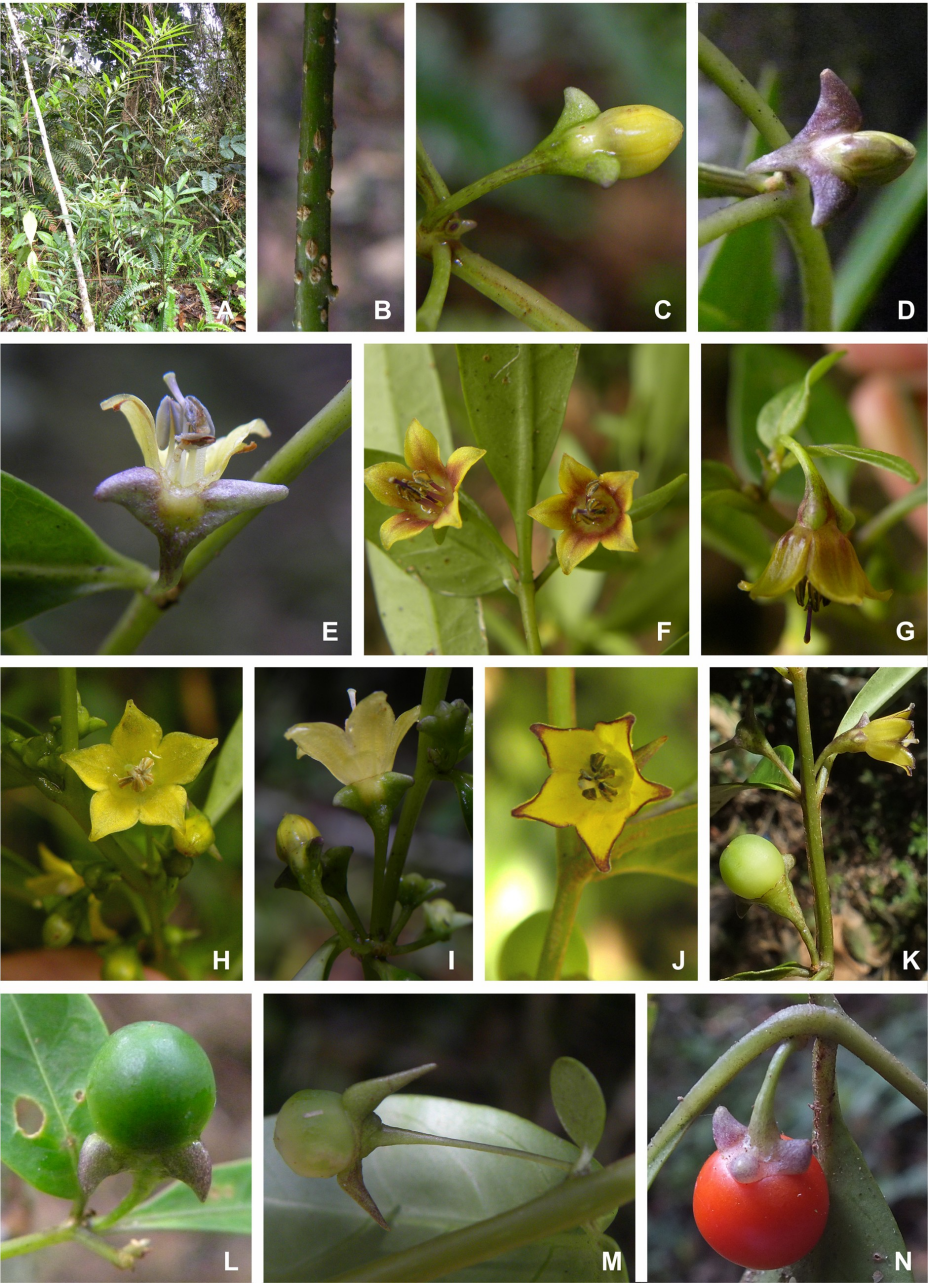
*Capsicum longifolium* Barboza & S. Leiva. (A) Plant. (B) Internode with lenticels. (C, D) Flower buds. (E) Flower, longitudinal section. (F) Flowers showing corolla yellow with brownish center. (G) Same flower as in F, lateral view. (H, I) Flowers with completely yellow corollas, upper and lateral view, respectively. (J, K) Flowers with yellow corollas with red-brown edges, upper and lateral view, respectively. (L, M) Immature fruits. (N) Mature fruit. Photos by S. Leiva González and G. E. Barboza.

**Diagnosis**. Similar to *Capsicum dimorphum* (Miers) Kuntze but differing in the long and narrow coriaceous leaves, the number of flowers (3–9), the unequal thick calyx appendages, and the glabrous vegetative organs and calyx.

**Description**. Scandent shrubs (0.60) 1.40–3 m tall, laxly branched, slightly plagiotropic. Young stems green, fragile, glabrous, striate with abundant ovoid and light dark lenticels; bark of older stems dark green, glabrous, striate with sparse white lenticels. Sympodial units difoliate, geminate, the leaf pair markedly anisophyllous in size and shape. Leaves simple, coriaceous, slightly discolorous, adaxial surface dark green and shiny, abaxial surface light green and opaque, glabrous on both surfaces and margins; the larger leaves with blades (7) 8.5–17 (18) cm long, (0.8) 1–2.5 cm wide, narrowly elliptic (ratio 6–10.8), major veins (11)13-17 on each side of midvein, base asymmetric and attenuate, margin entire, apex acuminate; petioles 0.2 (0.5–1.4) cm long, glabrous; the minor leaves 2.5–5.7 cm long, 1–2 cm wide (ratio 1.78–4), ovate or broadly elliptic, major veins 4–5 on each side of midvein, base short attenuate, sometimes asymmetric, margin entire, apex obtuse; petioles 0.1–0.5 cm long, glabrous. Flowers in fascicles of 3–7 (9) on a short shoot leaving evident scars when fallen, rarely solitary; flowering pedicels green, filiform, terete, pendent, slightly curved, not geniculate at anthesis, widening to the apex, 0.3–0.8 cm long, glabrous. Flower buds ovoid, yellow or purplish yellow. Calyx 2.5–3 mm long, 2.8–3 mm wide, cup-shaped, very thin, transparent, light green or greenish purple, the margin truncate, glabrous, with 2–3 thick appendages like triangular-compressed wings, 2–2.5 mm long, 1.8–2.2 mm wide, green or purple, glabrous. Corolla 6–8.5 mm long, 8–11 mm diam, stellate-campanulate, thick, entirely yellow or yellow with red-brown coloration at margin lobes or inside, without interpetalar tissue; tube (3) 4–5 mm long, glabrous inside and outside; lobes 3–3.5 (4) mm long, ca. 3 mm wide, broadly ovate, erect or patent, glabrous adaxially and abaxially, the tips papillose and cucullate. Stamens 5, equal, filaments equal, 2–2.6 mm long, white or red-brown, glabrous, inserted on the corolla ca. 2 mm from the base, with inconspicuous auricles at point of insertion; anthers 2–2.75 mm long, not connivent, elliptic, purplish white or brown. Ovary 1.6–1.8 mm long, 1.2 mm diam, subglobose, white or light green, glabrous; nectary 0.3–0.5 mm tall, white; style 5–5.8 mm long, white and lilac at the apex, widening distally, glabrous; stigma 0.3 mm long, 0.2–0.4 mm wide, light green, somewhat bilobed. Berry 0.8–1.3 cm diam, globose, slightly flattened at the apex, green when immature, orange at maturity, glabrous, not pungent, the pericarp lacking giant cells (endocarp smooth) and stone cells; fruiting pedicels 1–1.6 cm long, pendent, terete, widened distally; the fruiting calyx persistent, non-accrescent, 4–5.5 mm diam, discoid, green-purple or green, the appendages spreading or reflexed, short and wide (2–2.8 mm long, 2.4–2.6 mm wide at base) or long and more slender (4.5–5.5 mm long, ca. 1.5 mm wide at base), fleshy and subulate. Seeds ca. 24 per fruit, 1.7–2.3 mm long, 1.7–2.2 mm wide, not compressed, obconic, black, the surface reticulate, cells rectangular or polygonal in shape, lateral walls straight or slightly sinuate.

**Distribution and ecology**. Endemic to northern Peru (Amazonas, Cajamarca and Piura) and southern Ecuador (Zamora-Chinchipe) (Fig 2), growing in montane wet forests at mid elevations (1800–2200 m), associated with other Solanaceae shrubs (*Capsicum geminifolium* (Dammer) Hunz., *Solanum* spp., and *Deprea* spp.), *Cyathea* Sm. (Cyatheaceae), *Miconia* Ruiz & Pav. (Melastomataceae), *Piper* L. (Piperaceae), *Ocotea* Aubl. (Lauraceae), *Anthurium* Schott (Araceae), amongst other shrubs and trees. It grows in the interior of primary forest in shady areas.

**Phenology**. Flowering and fruiting from December to August, and probably all year.

**Etymology**—The species epithet refers to the shape of the leaves, which are the longest and narrowest elliptic leaves known thus far in the genus.

**Species Conservation Assessment**. Following the IUCN Criteria (IUCN 2017), we suggest *C*. *longifolium* deserves a status of Endangered. The extent of occurrence is calculated to be 19207 km2 (Criterion B1 < 20000 km2, Vulnerable), the area of occupancy, 24 km2 (Criterion B2 < 500 km2, Endangered). Although the species has been collected many times in the last 12 years, in San Francisco Biological Reserve (SFBR, Zamora-Chinchipe, Ecuador), it is known from only other 5 locations (Criterion B1a ≤ 10, Vulnerable), in areas not included in a National System of Protected Areas which would indicate a risk to the quality of its habitat (Criterion B2b).

**Fig 5:**
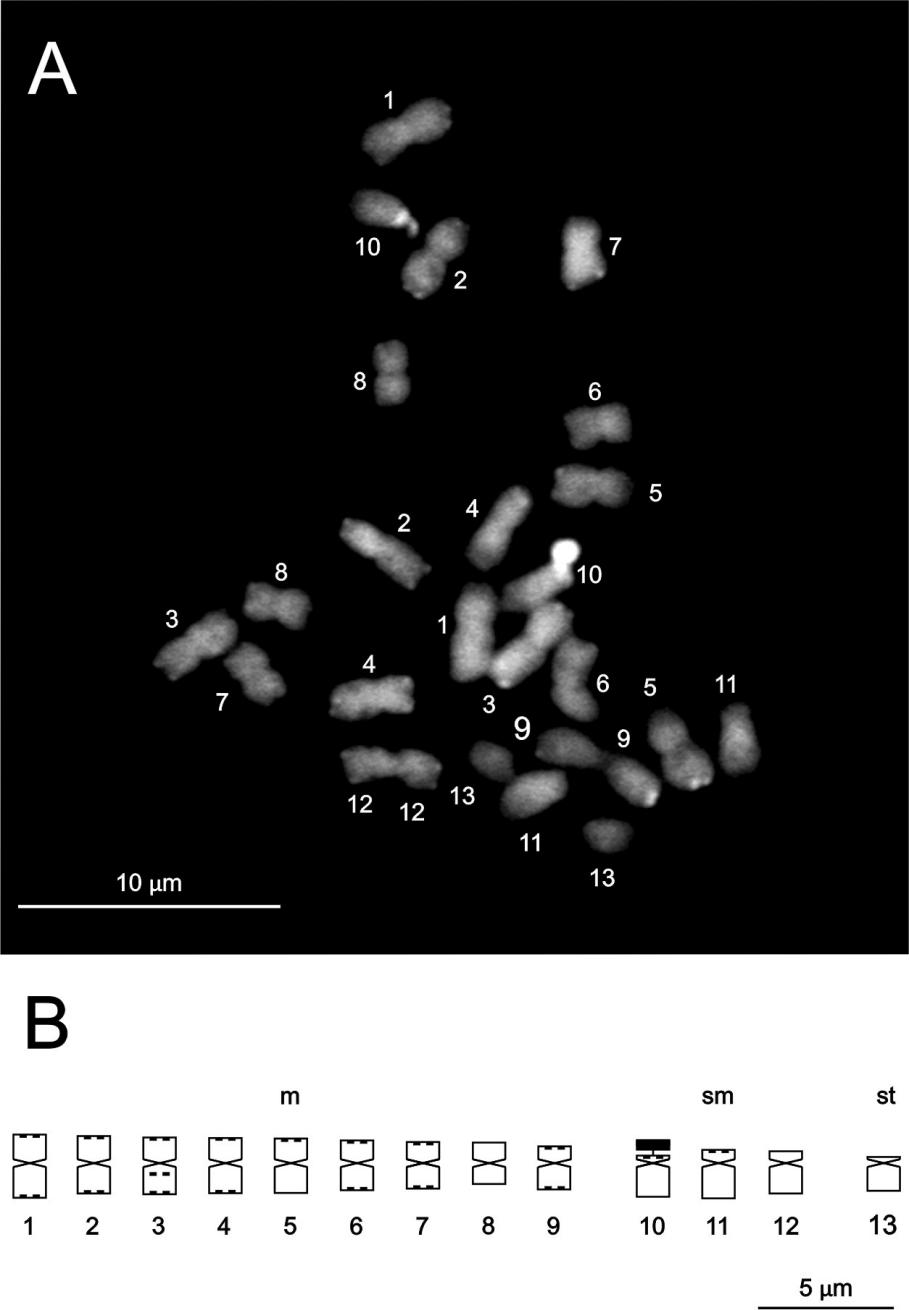
Somatic metaphase chromosomes and ideogram of *Capsicum longifolium*. (A) Methaphase chromosomes. (B) Ideogram. Solid black blocks or dots denote CMA+/DAPI- (NOR) or CMA+/DAPIo (terminal and intercalary) heterochromatic bands. The NOR is indicated as a separate block.

**Karyology**. This taxon possesses a 2n = 2x = 26 karyotype with 9 m pairs (1–9) of decreasing but rather similar size, 3 sm pairs (10–12), and one st pair (13) (Fig 5, S1 Table). Pair 10 (sm) is satellited. Two types of constitutive heterochromatin are found in this taxon, GC-rich heterochromatin (CMA+/DAPI-) and moderately GC-rich heterochromatin (CMA+/DAPIo). The fluorescent banding pattern is quite simple, with most of the chromosomes having similar-sized small terminal bands, except for pairs 8, 12 and 13, which are not banded, an intercalary band on the long arm of pair 3, all of them carrying moderately GC-rich heterochromatin, and the large heterochromatic nucleolar organizer region (NOR)-associated block on the short arm of satellite pair 10, bearing GC-rich heterochromatin (Fig 5, S4 Table).

**Fig 6:**
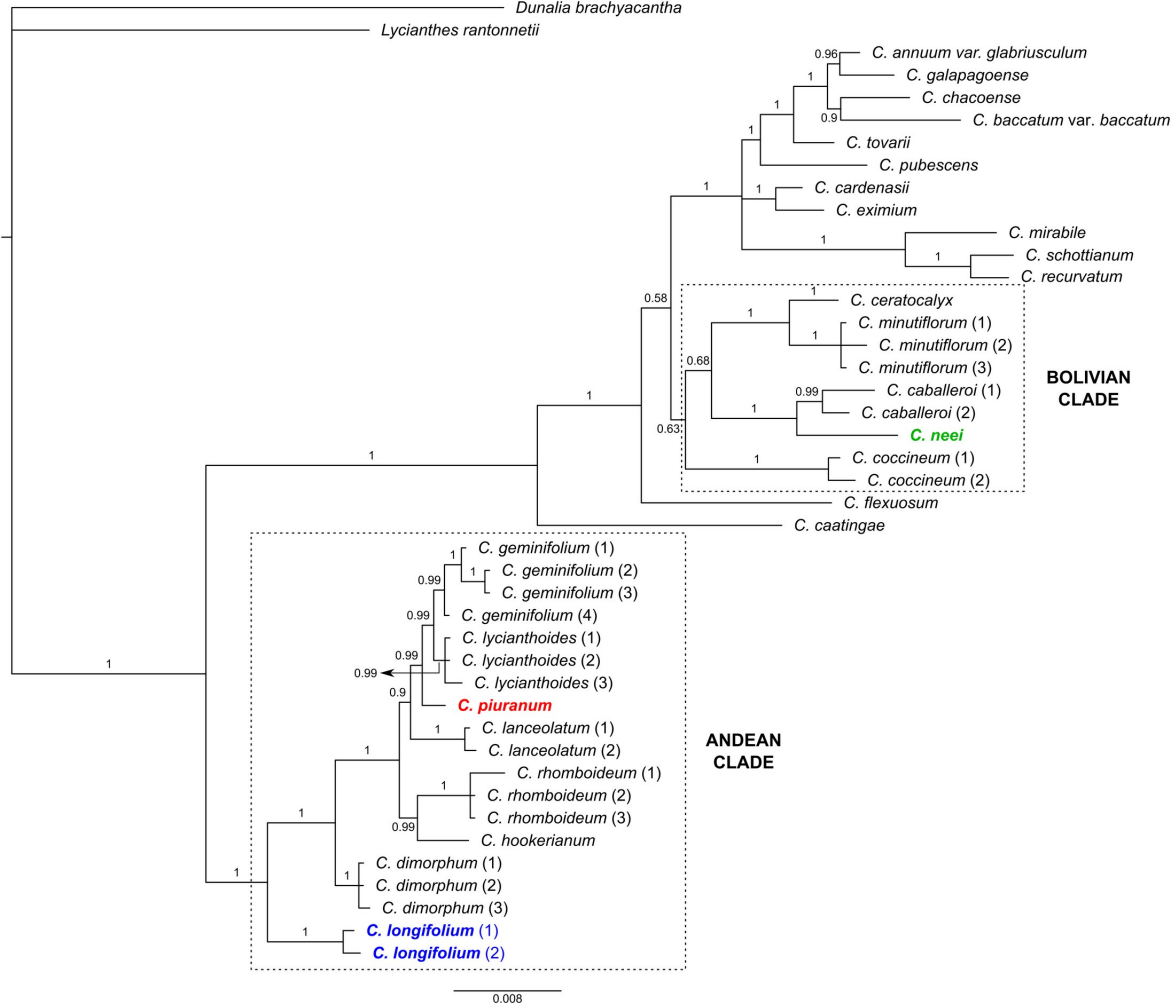
Bayesian majority-rule consensus tree of *Capsicum*. Posterior probabilities values indicated by each branch. New species are highlighted in bold-colored letters and the clade to which they belong is indicated.

**Affinities**. *Capsicum longifolium* is strongly resolved within the Andean clade, as the first diverging branch within the clade, sister to the rest of the species included in it (Fig 6).

**Additional specimens examined**. **PERU. Amazonas**: Rodríguez de Mendoza, Omia, entre la Cordillera y Quebrada de Agua Sal, 06°26'18''-06°25'4.4''S, 77°10'30.8''-77°10'2.3''W, 2457–2118 m, 22 Sept 2008 (fl, fr), *V*. *Quipuscoa S*. *et al*. *4374* (HUSA, HUT, F, USM). **Cajamarca**: San Ignacio, Huarango, Quebrada El Mirador, 05°16'12"S, 78°40'01"W, 2200 m, 13 Mar 2000, *J*. *Campos et al*. *6607* (MO). **Piura**: Huancabamba, distr. Carmen de la Frontera, Río Samaniego, margen derecha, zona de amortiguamiento del Santuario Nacional Tabaconas-Namballe, 2150–2200 m, 25 Apr 2003 (fl, fr), *S*. *M*. *Baldeón et al*. *5316* (USM). **ECUADOR. Zamora-Chinchipe**: Estación Biológica San Francisco (EBSF), camino hacia la antena, pasando el río San Francisco, 3°58’22.5”S, 79°04’40.9”W, 1830 m, 2 May 2017(fl), *G*. *E*. *Barboza & S*. *Leiva González 4821* (CORD); at the same place, *S*. *Leiva González 6531* (HAO); EBSF, a unos 300 m después del cruzar el Río San Francisco, por el camino del Atajo, 3°58’21.6”S, 79°04’41.4”W, 1839 m, 17 Aug 2017 (fl, fr), *G*. *E*. *Barboza & S*. *Leiva González 4846 & 4851* (LOJA, duplicates to be sent to CORD & HAO); EBSF, después de cruzar el Río San Francisco, 3°58’22.5”S, 79°04’39.3”W, 1888 m, 17 Aug 2017 (fl, fr), *G*. *E*. *Barboza & S*. *Leiva González 4849 & 4850* (LOJA, duplicates to be sent to CORD); road Loxa-Zamora, 5 km W of Tambo, 2100 m, 14–19 Jul 1959 (fl), *G*. *Harling 5867* (S); above Valladolid on road to Yanganá, 2700 m, 2 Feb 1985 (fl), *G*. *Harling & L*. *Andersson 21464* (GB).

*Capsicum longifolium* is unique in the genus in having the longest and narrowest leaves and the striking calyx appendages that arise from the calyx tube as lateral compressed thick expansions or wings (Fig 4C–4E and 4L–4N). Apart from that, it is morphologically most similar to *C*. *dimorphum* with which it shares the shape and color of the corolla, fruit and seeds. *Capsicum longifolium* can be distinguished by having completely glabrous vegetative organs and calyces, long and narrow (ratio 6–10.8) coriaceous major leaves, flowers in fascicles of 3–7 (9) on a short shoot and calyces with 2–3 thick appendages like triangular-compressed wings compared to the pubescent vegetative organs and calyces, the shorter and wider (ratio 4–5.25) membranaceous major leaves, the solitary or up to 5 axillary flowers, and the toothless calyx or with 3 tiny appendages of *C*. *dimorphum*. Another species of *Capsicum* sympatric with *C*. *longifolium* (especially in SFBR, Ecuador) is *C*. *geminifolium* that has a dense indumentum, long apiculate leaves, longer pedicels (5 cm long), thin calyx appendages, and funnel-shaped yellow corollas with many purple or maroon spots inside.

Variation in corolla color and length of the fruiting calyx appendages can be observed in the field in individuals growing under the same environmental conditions. The corolla is mainly pure yellow (Fig 4E, 4H and 4I), but occasional specimens have corolla lobes red-to brown-edged (Fig 4J and 4K), or with a red-brown ring inside the corolla limb (Fig 4F and 4G); in this latter case, the filaments and the style are also red-brown. In general, the fruiting calyx appendages do not enlarge considerably (Fig 4L and 4N) but some specimens have long appendages (Fig 4M).

The chromosome number 2n = 26 found in *C*. *longifolium* is the same as that of *C*. *rhomboideum* (Dunal) Kuntze [32], *C*. *lanceolatum* (Greenm.) C.V. Morton & Standl. [33] and *C*. *lycianthoides* Bitter [34], all belonging to the Andean clade. Their karyotype formulas are quite similar, but that of *C*. *longifolium* is closest to *C*. *lycianthoides* (9 m + 3 sm + 1 st) than to *C*. *rhomboideum* (10 m + 1 sm + 2 st). The species of this clade share small amounts of heterochromatin, a single pair of NOR, short karyotype lengths, and small chromosomes in comparison with other species of the genus [32]. The karyotype of *C*. *longifolium* is almost half the length of *C*. *rhomboideum*, the latter with the shortest karyotype length known until now for the entire genus.

***Capsicum piuranum* Barboza & S. Leiva, sp. nov.** [urn:lsid:ipni.org:names: 77192559–1]

Type: Peru. Piura: Prov. Huancabamba, borde de carretera y riachuelo, 5°22’46”S, 79°33’47”W, 2311–2459 m, 22 Mar 2011 (fl, fr), *T*. *Mione 812* (holotype, CORD [CORD-00006936]; isotype, NY [NY-03231447]).

Figs 7 and 8

**Fig 7:**
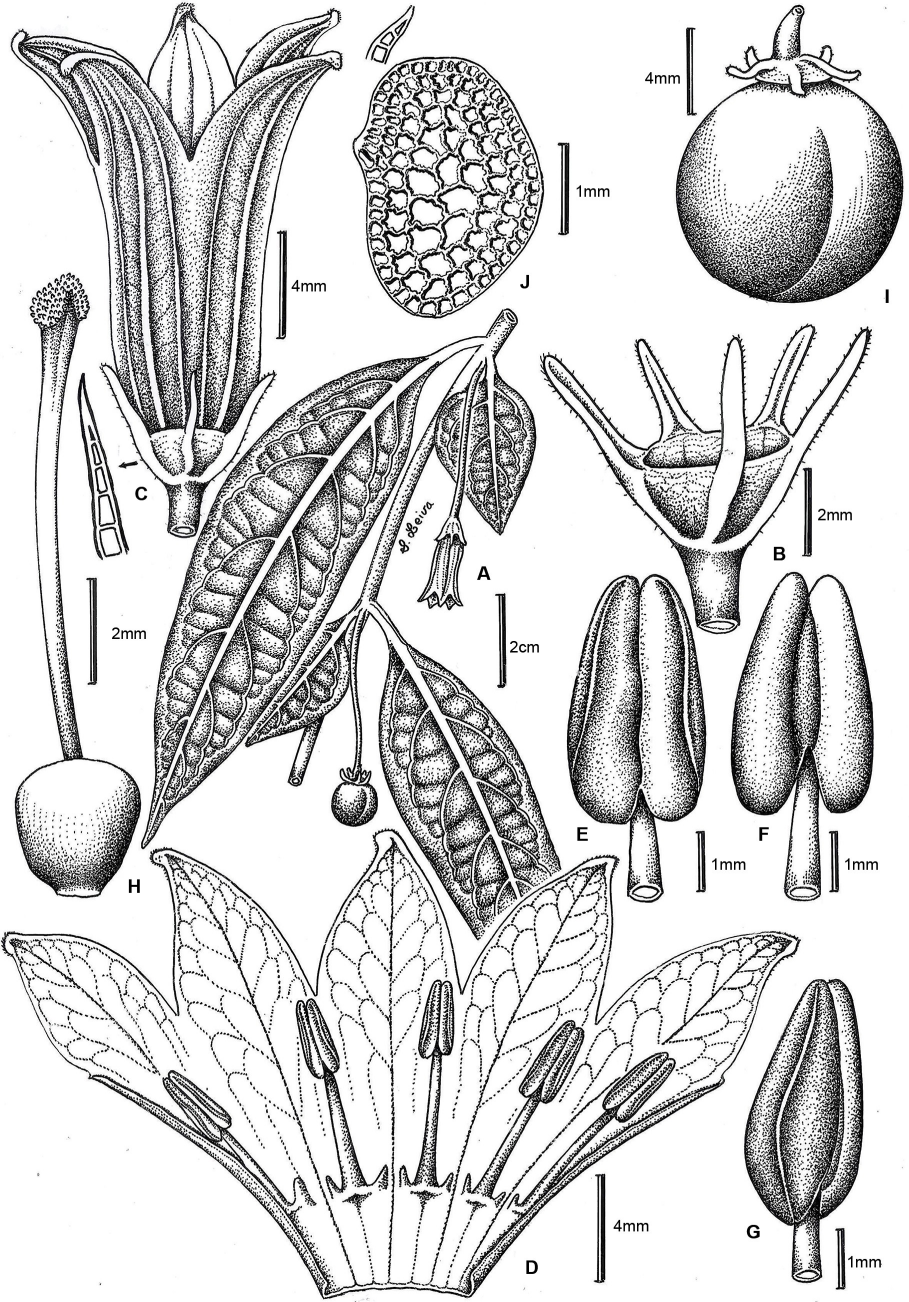
*Capsicum piuranum* Barboza & S. Leiva. (A) Flowering branch. (B) Calyx. (C) Flower. (D) Opened corolla. (E, F, G). Anther, ventral, dorsal and lateral view, respectively. (H) Gynoecium. (I) Fruit. (J) Seed. Drawn by S. Leiva González.

**Fig 8:**
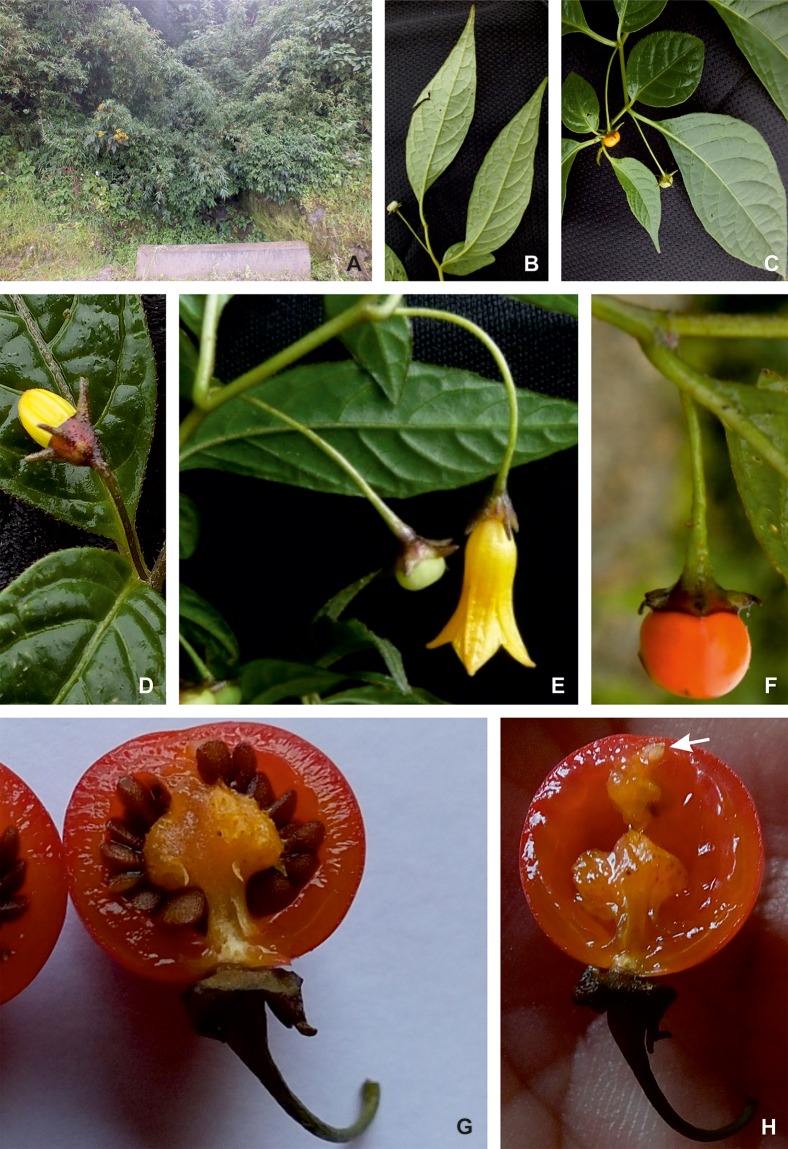
*Capsicum piuranum* Barboza & S. Leiva. (A) Plant. (B) Leaves, abaxial surface. (C) Fruiting branch. (D) Flower bud. (E) Flower and immature fruit. (F) Mature fruit. (G) Fruit, transverse section, showing placenta and seeds. (H) Fruit transverse section, showing a stone cell at the apex (arrow). Photos by S. Leiva González and G. E. Barboza.

**Diagnosis**. Like *Capsicum caballeroi* M. Nee but differing in the purple calyx, the 5 equal calyx appendages, the longer tubular-campanulate corolla, the globose orange non-pungent mature fruit, and the black seeds.

**Description**. Scandent shrubs 2–2.20 (3) m tall, densely branched. Young stems green, shiny, fragile, flexuous, glabrous, striate; bark of older stems green to dark brown, glabrous, striate; lenticels absent. Sympodial units difoliate, geminate, leaf pair markedly anisophyllous in size and shape. Leaves simple, membranaceous, discolorous, adaxial surface dark green and shiny, abaxial surface light green and opaque, glabrous or with sparse simple antrorse trichomes 0.5–1.2 mm long adaxially and abaxially, occasionally trichomes more abundant on main veins and margins; the larger leaves with blades (8) 12–17.7 cm long, (2) 2.5–4.5 cm wide, elliptic, major veins 7–9 on each side of midvein, base asymmetric and attenuate, margin entire, apex long-acuminate; petioles 0.7–1.4 (1.7) cm long, slightly winged from the decurrent leaf bases, glabrous or glabrescent with trichomes like those of the leaves; the minor leaves 2.5–4.5 cm long, 1.5–2.6 cm wide, ovate or elliptic, major veins 3–4 on each side of midvein, base short attenuate or rounded, asymmetric, margin entire, apex acute or slightly rounded; petioles 0.2–0.5 cm long, glabrescent or pubescent. Flowers solitary or in fascicles of 3; flowering pedicels green, filiform, terete, pendent, slightly curved, not geniculate at anthesis, 1.9–2.6 cm long, glabrous or glabrescent, the trichomes simple, non-glandular, multicellular, antrorse, 0.30–0.45 mm long. Flower buds ovoid, yellow or pale yellow. Calyx 1.5–2.6 (3) mm long, 3–4 mm wide, cup-shaped, thick, purple or greenish purple, the margin truncate, glabrescent to pubescent, with 5 appendages (0.9) 2.5–3 mm long, 0.5–0.8 mm wide, thick, erect, subulate, inserted close to the margin, glabrous or glabrescent with the same trichomes as pedicels and calyx tube. Corolla 14.5–17 mm long, 12–17 mm diam, tubular-campanulate, thick, entirely yellow; tube 11–12 mm long, glabrous inside and outside; lobes 3.5–5 mm long, 4.5–5 mm wide, broadly ovate, erect, glabrous adaxially and abaxially, the tips papillose. Stamens 5, equal, filaments subequal, 3–5 mm long, greenish white, glabrous, inserted on the corolla 3–4 mm from the base, with inconspicuous auricles at point of insertion; anthers 2–2.5 (2.8) mm long, slightly connivent before anthesis, elliptic, yellowish white. Ovary 1.25–1.5 mm long, 1.5 mm diam, subglobose, white, glabrous; nectary ca. 0.5 mm tall, inconspicuous, yellowish white; style 7.5–8 mm long, white, widening distally, glabrous; stigma 0.5 mm long, 0.8–1 mm wide, green, somewhat bilobed. Berry 0.9–1.2 cm in diameter, globose, slightly flattened at the apex, green or white when immature, orange to red at maturity, glabrous, not pungent, the pericarp lacking giant cells (endocarp smooth), sclerotic granules 2, polyhedral, yellowish white; fruiting pedicels 2.8–3.6 cm long, pendent, slightly striate and widened distally; the fruiting calyx persistent, non-accrescent, ca. 4 mm diam, discoid, green-purple or green, the reflexed appendages 5–6.1 mm long, 0.8–1 mm wide at base, fleshy and subulate. Seeds ca. 50–80 per fruit, 2–2.2 mm long, ca. 2.5 mm wide, somewhat compressed, subreniform or obconic, dark brown, the surface reticulate, cells polygonal in shape, lateral walls straight or slightly sinuate.

**Distribution and ecology.** Endemic to a restricted area in northern Peru (Piura, Fig 2) growing in montane misty rain forests, associated with other Solanaceae shrubs (*Solanum* spp. and *Streptosolen jamesonii* (Benth.) Miers), *Begonia* L. (Begoniaceae), *Otholobium* C.H.Stirt. (Fabaceae), *Aphelandra* R. Br. (Acanthaceae), *Juglans* L. (Juglandaceae) amongst other herbs and shrubs. It grows in margins of forest and near streams, between 2300–2860 m elevation, in areas of low temperature and rich soils.

**Phenology.** Flowering from November to May, with a peak of fruiting in March–May.

**Etymology.** The new species is named in allusion to its very restricted habitat in Department Piura (Peru).

**Species Conservation Assessment.** According to IUCN criteria (IUCN, 2017), *C*. *piuranum* is proposed as Critically Endangered (CR) species. The extent of occurrence is calculated to be 10.195 km2 (Criterion B1 < 100 km2, Critically Endangered), the area of occupancy, 8 km2 (Criterion B2 < 10 km2, Critically Endangered). The species is known from only three locations (Criterion B2a ≤ 5, Endangered) and the number of mature individuals observed in each subpopulation is ≤ 50 (Criterion C2a, Critically Endangered).

**Fig 9:**
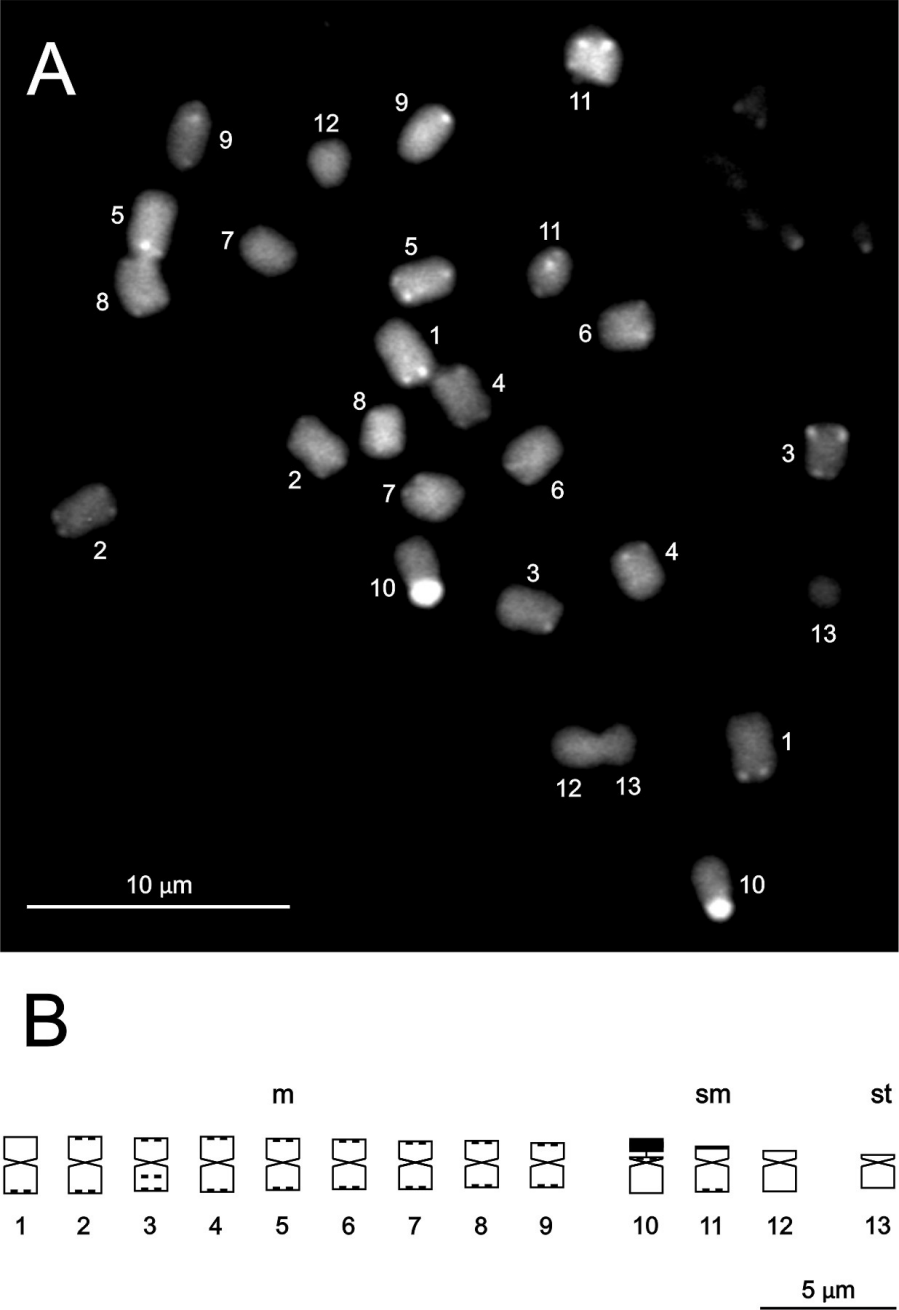
Somatic metaphase chromosomes and ideogram of *Capsicum piuranum*. (A) Methaphase chromosomes. (B) Ideogram. Solid black blocks or dots denote CMA+/DAPI- (NOR) or CMA+/DAPIo (terminal and intercalary) heterochromatic bands. The NOR is indicated as a separate block.

**Karyology.** A somatic chromosome number of 2n = 2x = 26 was found in this species. The karyotype comprises 9 m pairs of rather similar length (1–9), 3 sm pairs (10–12), and one st pair (13) (Fig 9, S1 Table). One pair is satellited (10 sm). As in *C*. *longifolium*, this species bears two types of constitutive heterochromatin, GC-rich heterochromatin (CMA+/DAPI-) located in the large heterochromatic band associated to the NOR in pair 10, and moderately GC-rich heterochromatin (CMA+/DAPIo), located in the small terminal bands and in the intercalary band on the long arm of pair 3. The fluorescent banding pattern is quite simple and very similar to *C*. *longifolium*, except for the presence of 3 small bands that are not seen in that species (Fig 9, S4 Table).

**Affinities**. *Capsicum piuranum* is resolved within the Andean clade, strongly supported as sister to the *C*. *lycianthoides*-*C*. *geminifolium* assemblage (Fig 6).

**Additional specimens examined. PERU. Piura:** Prov. Huancabamba, carretera Canchaque-Huancabamba, km 98–99 (ca. 20 km de Canchaque rumbo a Huancabamba), subiendo a Cuello del Indio, 05°22’43”-05°22’44”S, 79°33’34”-79°33’37”W, 2341–2346 m, 8 May 2017 (fl, fr), *G*. *E*. *Barboza & S*. *Leiva González 4841 & 4842* (CORD); carretera Huancabamba-Canchaque, a una hora del Abra Cruz Blanca, 05°22'25"S, 79°34'02"W, 2860 m, 13 Apr 2017 (fl, fr), *M*. *Cueva 2912* (USM); distrito Canchaque, km 98–99 (ruta Canchaque-Huancabamba), 5° 22’44.1”-5° 22’42.7”S, 79° 33’37.2”-79° 33’34.1” W, 2341–2346 m, 8 May 2017 (fl, fr), *S*. *Leiva González & G*. *E*. *Barboza 6561 & 6562* (HAO); Prov. Morropon: Chalaco, Bosque Mijal, 05°03'51.1"S, 79°43'25.9"W, 2800 m, 1 Nov 2015 (fl, fr), *M*. *Cueva et al*. *2655* (USM); same locality and date, *M*. *Cueva et al*. *2656*, *2657 & 2658* (USM, duplicates to be sent to CORD, HUSA).

*Capsicum piuranum* is morphologically most similar to *C*. *caballeroi* M. Nee of the Bolivian yungas (Santa Cruz and Cochabamba) based on their campanulate yellow corollas. However, these species can be distinguished in the calyx color, the calyx appendages (number, size, and shape), the position of the corolla lobes at anthesis, the fruit size, shape, color and pungency, the presence of stone cells, and the seed color. *Capsicum piuranum* has a purple or greenish purple calyx with 5 equal subulate appendages (Fig 8D and 8E), while *C*. *caballeroi* has green calyx with 10 unequal linear appendages. Corolla lobes are erect compared to those of *C*. *caballeroi* which are recurved. Mature fruits are smaller (up to 1.2 mm diam), globose, orange and not pungent in *C*. *piuranum* but are larger (up to 1.6 mm diam), globose-depressed to globose, bright red and pungent in *C*. *caballeroi*. *Capsicum piuranum* has two stone cells (Fig 8H) and dark brown smaller seeds (2–2.2 mm long, ca. 2.5 mm wide) while *C*. *caballeroi* lacks of stone cells and the seeds are pale yellow or light brown and larger (3.2–4 mm long, 3.8–5 mm wide).

*Capsicum piuranum* is sympatric with other two Andean species, *C*. *geminifolium* (Dammer) Hunz. and *C*. *rhomboideum*, both of which have also yellow corollas and non-pungent fruits, but a moderate to dense pubescence on stems and leaves. *Capsicum geminifolium* differs in having longer calyx appendages (3–6.5 mm long) compared to *C*. *piuranum* (2.5–3 mm long) and funnel-shaped generally purple spotted yellow corollas (tubular-campanulate and pure yellow in *C*. *piuranum*, Fig 8E). *Capsicum rhomboideum* has ovate or rhomboid-ovate leaves, up to 12 axillary flowers, campanulate-rotate smaller corollas (0.6–0.95 cm long), and smaller (up to 0.9 cm diam) bright red to blackish red fruits in contrast to *C*. *piuranum* where leaves are elliptic or narrowly elliptic (sometimes the minor leaves are ovate, Fig 8B and 8C), the flowers are solitary or in fascicles of 3 (Fig 8E), the corolla is tubular-campanulate and longer (14.5–17 mm long), and the fruits are larger (0.9–1.2 cm diam) and orange colored.

This species exhibits the same number of chromosomes as *C*. *longifolium* and the species that belong to the Andean clade [32–34]. In addition, *C*. *piuranum* and *C*. *longifolium* share the same karyotype formula, little heterochromatin, one only pair of NOR, and the smallest chromosomes in the genus [32].

The markedly anisophyllous leaves, the deflexed non-geniculate pedicels, the yellow corollas, the globose orange to red non-pungent fruits, the absence of giant cells and the presence of stone cells in the pericarp, the black seeds, and the chromosome number 2n = 26 place *C*. *piuranum* in the Andean clade proposed by Carrizo García et al. [1], as it has been determined in this work based on DNA data.

***Capsicum neei* Barboza & X. Reyes, sp. nov.** [urn:lsid:ipni.org:names: 77192560–1]. Type: Bolivia. Chuquisaca: Prov. Hernando Siles, a 4.1 km del puente nuevo de Monteagudo viniendo desde Monteagudo, sobre mano derecha, -19.804617 S, -64, 019923 W, 16 Dec 2017 (fl), *G*. *E*. *Barboza 4927* (holotype, LPB; isotypes, CORD [CORD-00006935, CORD-00006956], NY).

Figs 10 and 11

**Fig 10:**
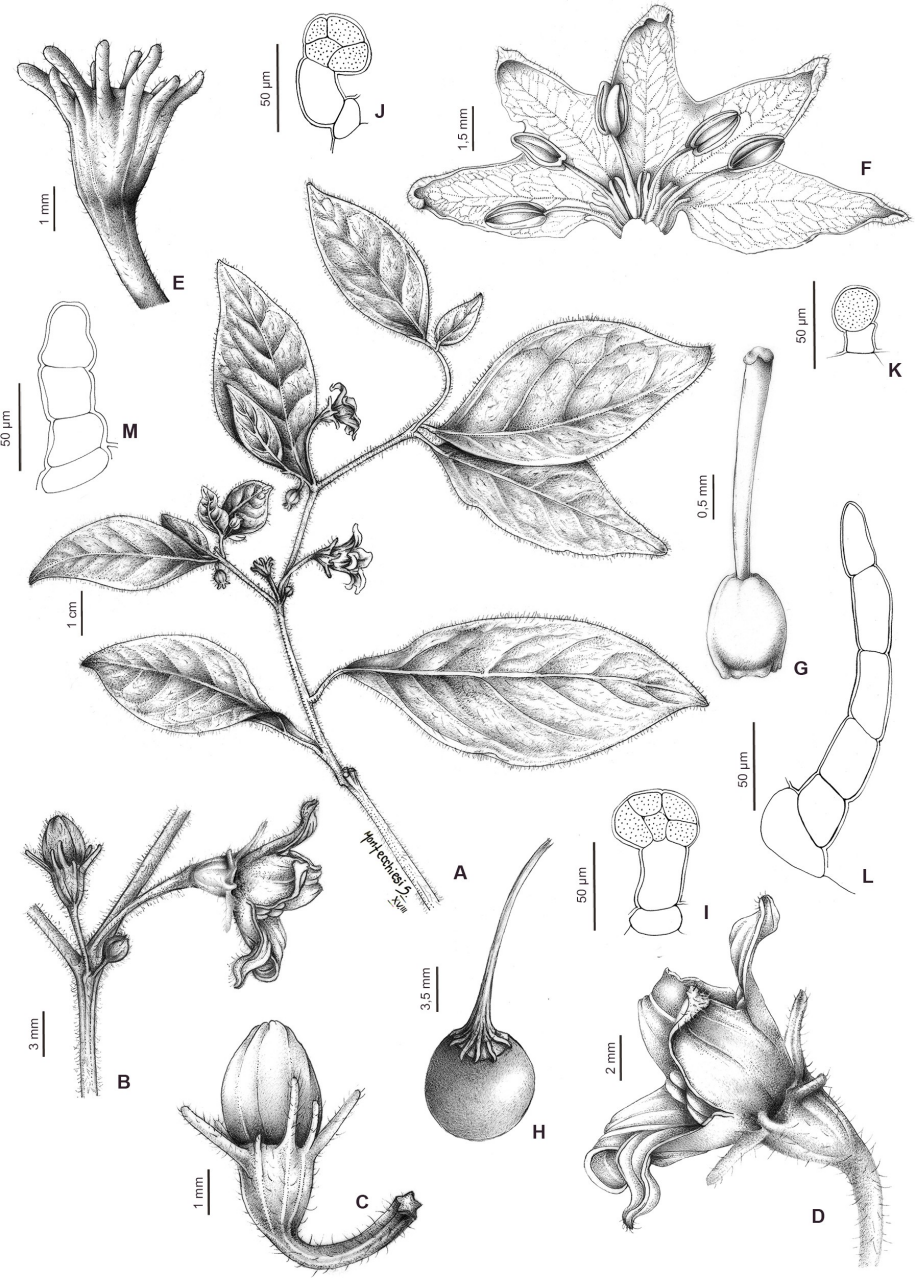
*Capsicum neei* Barboza & X. Reyes. (A) Flowering branch. (B) Inflorescence. (C) Flower bud. (D) Flower. (E) Calyx. (F) Opened corolla. (G) Gynoecium (H) Fruit. (I) Glandular trichome of the inside calyx. (J) Glandular trichome of the pedicels. (K) Glandular trichome of the inside corolla. (L) Non-glandular trichome of the outside calyx. (M) Non-glandular trichome of the outside corolla lobes. Drawn by S. Montecchiesi.

**Fig 11:**
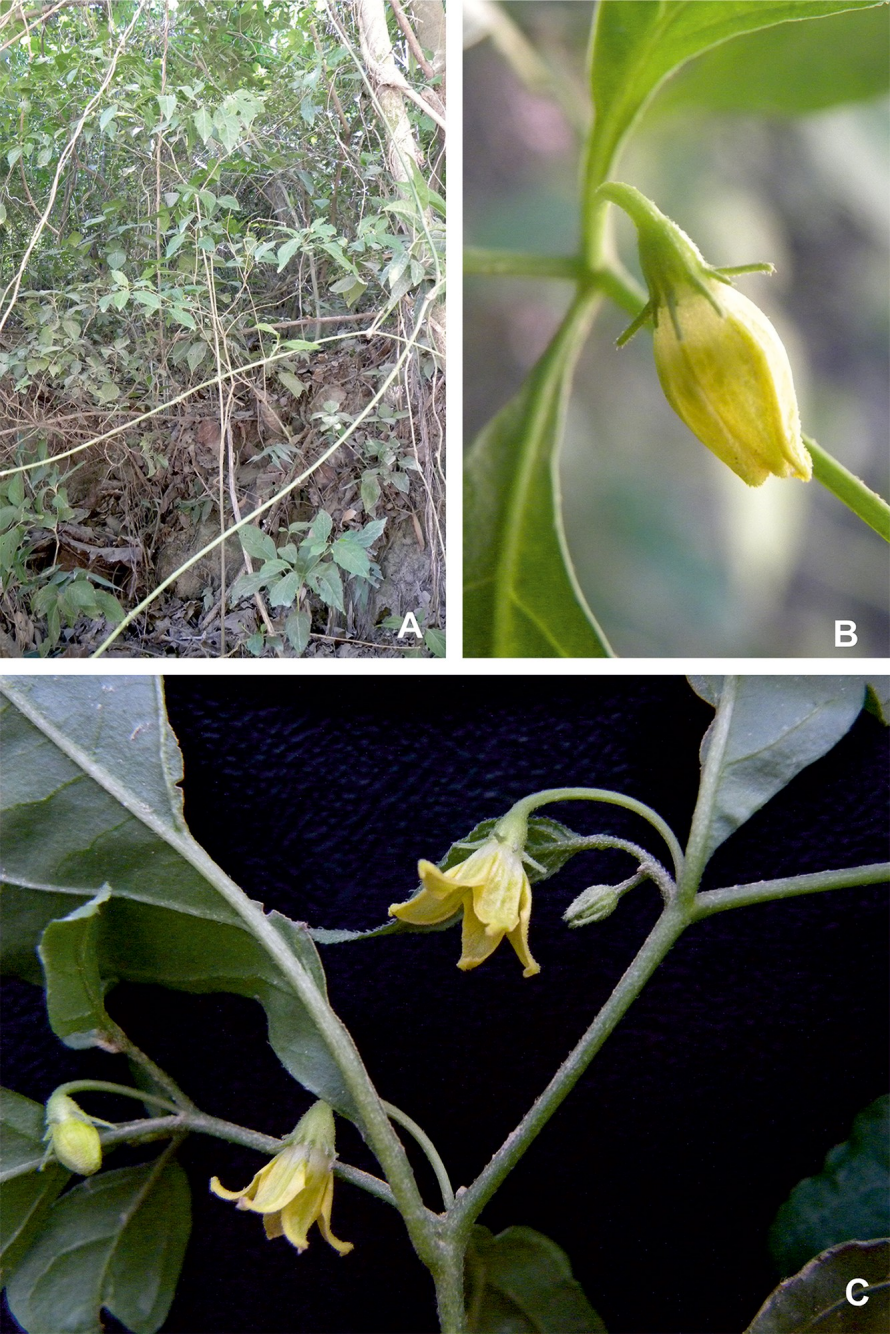
*Capsicum neei* Barboza & X. Reyes. (A) Plant. (B) Flower bud. (C) Flowering branch. Photos by G. E. Barboza.

**Diagnosis**. Like *Capsicum minutiflorum* Rusby (Hunz.) but differing in the non-geniculate pendent flowering pedicels and the strongly nerved calyx with 10 unequal appendages.

**Description**. Small shrubs 0.70–2 (3) m tall, thin, erect, laxly branched above. Young stems green, slim, fragile, glabrescent, and slightly striate, without lenticels; bark of older stems light brown, glabrous, with a few oblong lenticels. Sympodial units difoliate, geminate, leaf pair not markedly anisophyllous in size and shape. Leaves simple, membranaceous, glabrescent on both surfaces and margins with 4-7-celled non glandular trichomes 0.2–0.5 mm long; the larger leaves with blades (5.5) 6.7–11 cm long, 2.1–4 (4.5) cm wide, elliptic or ovate, major veins 3–4 on each side of midvein, base attenuate, margin entire, apex acute; petioles 0.3–0.8 (1.5) cm long; the minor leaves 2.7–4.6 (6) cm long, 1.2–1.8 (2.3) cm wide, elliptic or ovate, major veins 2–3 on each side of midvein, base attenuate, margin entire, apex obtuse or acute; petioles 0.2–0.5 (0.8) cm long, with similar pubescence as in larger leaves. Flowers 2–4 per axil, rarely solitary; flowering pedicels green, filiform, striate, pendent, slightly curved, not geniculate at anthesis, (0.65) 0.8–1.5 cm long, with sparse 5-6-celled non-glandular trichomes and tiny dark glandular trichomes (stalk unicellular, head multicellular). Flower buds ovoid, greenish pale yellow. Calyx 1.7–2.5 mm long, 2–3 mm wide, cup-shaped, green, with 10 nerves clearly evident, the margin truncate, pubescent, with non-glandular trichomes 0.3–0.6 mm long outside and dense glandular pubescence inside (head multicellular, stalk unicellular), 10 unequal linear appendages, green, the five longer appendages (0.7) 0.9–1.75 (2) mm long, emerging almost from the margin, the five shorter 0.2–0.8 (1.2) mm long, emerging 0.8–1 mm below the margin, with the same non glandular trichomes of the calyx tube. Corolla (6) 8–10 mm long, 5–6 mm diam, stellate, delicate, entirely yellow or with small brownish green spots in the base of the lobes and tube inside, with a thin interpetalar tissue; tube 3–4.5 mm long, with tiny glandular trichomes (head and stalk one celled each) inside and glabrescent outside; lobes 3.5–5.5 mm long, ca. 2 mm wide, ovate, erect, glabrous adaxially and with sparse non-glandular trichomes abaxially, the tips papillose and cucullate. Stamens 5, subequal, filaments 1.4–1.75 mm long, cream, glabrous, inserted on the corolla ca. 1.2 mm from the base, with inconspicuous auricles at point of insertion; anthers (1.5) 1.8–2 mm long, not connivent, elliptic, light yellow. Ovary ca. 1.2 mm long, 1.3 mm diam, ovoid or subglobose, light green, glabrous; nectary ca. 0.3 mm tall, style 3.75 mm long, cream, widening distally, glabrous; stigma ca. 0.2 mm long, 0.3 mm wide, light green, somewhat bilobed. Berry 0.4–0.75 cm diam, globose, green when immature, red at maturity, glabrous; fruiting pedicels (1.3) 1.8–2.3 cm long, pendent, striate and widened distally; the fruiting calyx persistent, non-accrescent, ca. 4 mm diam, discoid, the appendages spreading 1–2 mm long, subulate. Seeds unknown.

**Fig 12:**
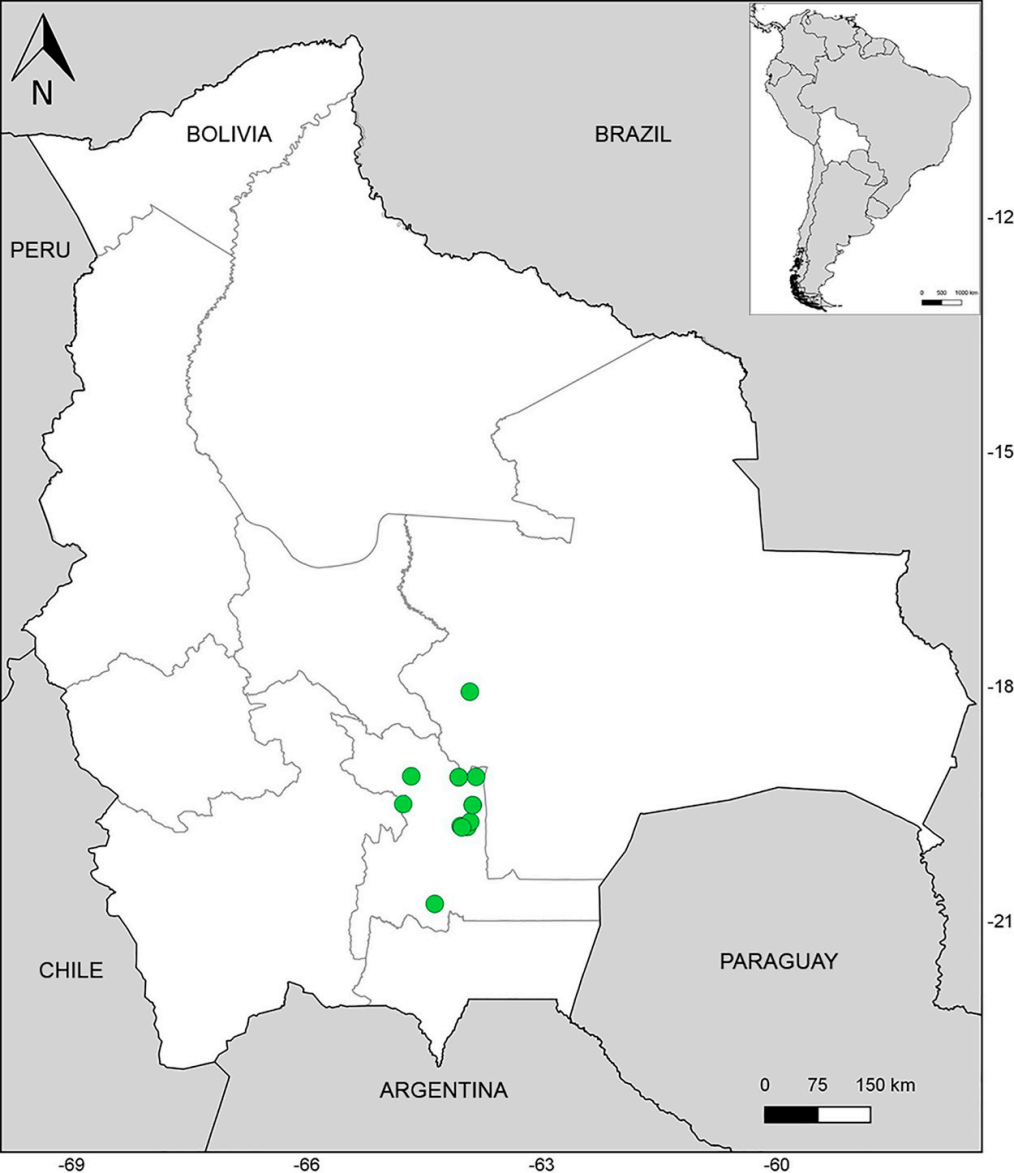
Distribution of *Capsicum neei* Barboza & X. Reyes.

**Distribution and ecology.** Endemic to southeastern Bolivia (Fig 12), mainly in the Serranías Iñao, Yahuañanca and Khaskha Orkho (Dpt. Chuquisaca). A few collections have been recorded from the Yungas (Dpt. Santa Cruz). *Capsicum neei* is most commonly collected in the Boliviano-Tucumano Forest in both Departments [35] from understories at the foot of cloud forest hillsides and deciduous forests, between 1100–1750 m elevation. It grows associated with members of Juglandaceae, Lauraceae, Myrtaceae, Leguminosae, ferns and bryophytes.

**Phenology**. Flowering and fruiting from October to May.

**Etymology.** The epithet is in honor to Dr. Mike Nee (NY), a solanaceous specialist who carried out extensive explorations in the Bolivian territory and separated specimens of this species as a rare or probable new species in various herbaria.

**Species Conservation Assessment.** According to IUCN criteria [15], *C*. *neei* is proposed as Near Threatened species. The species meets the area requirements under criterion B for threatened (EOO: 16912 km2, B1 < 20000 km2, Vulnerable; AOO: 44 km2, B2 < 500 km2, Endangered) and is declining, but the population is not severely fragmented and occurs in more than 10 locations. *Capsicum neei* has been collected many times in the last 23 years in a recently Protected Area: National Park and Integrated Management Natural Area “Serranía Iñao” [36], and in nearby areas which suggests that both the decline in its geographic range (EOO and AOO) and the population size will not be significantly affected in the forthcoming years.

**Affinities**. *Capsicum neei* is nested within the Bolivian clade, strongly resolved as sister to *C*. *caballeroi* (Fig 6).

**Additional specimens examined. BOLIVIA. Chuquisaca**: Hernando Siles, ca. 7 km de Monteagudo, inicio del cañón Heredia, 19°47'17''S, 64°02'08''W, 1127 m, 13 Dec 2006 (fl, fr), *H*. *Huaylla et al*. *2178* (HSB, MO); Parque Nacional y área natural de manejo integrado de la Serranía del Iñao, cuenca del río Limón, 19°44'01"S, 63°54'52"W, 1247 m, 15 Dec 2006 (fr), *E*. *Portal et al*. *108* (HSB, MO); foot of Cerro Urkhal path before 2nd river crossing, 19°48'S 63°57'W, 1300 m, 4 Oct 2000 (fl), *K*. *Wendelberger 170* (HSB, MO); Luis Calvo, Ticucha, serranía del Iñao, 12 km al NO de la comunidad de Ticucha, 19°35'0.4”S, 63°53'12.7”W, 1431 m, 11 Apr 2003 (fl), *A*. *Carretero et al*. *824* (HSB, MO, NY); Entierrillos, aprox. a 5 km de la escuela de Entierrillos, serranía del Iñao, 19°31'S, 63°52'W, 1700 m, 18 Dec 2003 (fl), *A*. *Carretero et al*. *939* (HSB, MO); Serranía del Iñao, pasando la Laguna, 19°31'S, 63°52'W, 18 Dec 2003 (fl), *A*. *Carretero et al*. *998* (HSB, MO, NY); Las Frías, ca. a la la cima de la serranía de Ñahuañanca, 19°09'30.6"S, 63°50'40.6"W, 1930 m, 22 Dec 2003 (fl, fr), *A*. *Carretero et al*. *1067* (HSB, MO, NY); Las Frías, ca. 1/2 km de la vivienda de Sr. Severino Daza, hacia la cima de la serranía de Yahuañanca, 19°09'31"S, 63°50'23"W, 1600 m, 23 Dec 2004 (fl), *A*. *Carretero et al*. *1085* (HSB, MO, NY); Sud Cinti, ca. 3 horas en caballo al NW de la comunidad de Orocote entre los ríos Limonal y Cochayo, 20°47'S, 64°21'W, 1650 m, 29 Apr 2005 (fr), *R*. *Lozano 1207* (HSB, MO); Tomina, aprox. 800 m. antes de llegar a Llantoj, de La Florida subiendo hacia el E de la Serranía de Kaska Orcko, 19°09'46"S, 64°03'42"W, 1750 m, 11 Oct 2004 (fl, fr), *J*. *Gutiérrez R*. *1004* (HSB, MO); Llantoj, aprox. 800 m antes de llegar a Llantoj, de la Florida subiendo hacia el E de la Serranía de Kaska Orcko, 19°09'46"S, 64°03'42"W, 1750 m, 15 Dec 2004 (fr), *J*. *Gutiérrez R*. *1072* (HSB, MO); Rio Limón Valley between Padilla and Monteagudo, 1500 m, 1 Jan 1995 (fl), *J*. *R*. *I*. *Wood 9104* (NY). **Santa Cruz.** Prov. Florida, Mairana, La Yunga de Mairana, 18°04’13”S, 63°55’08”W, 2190 m, 15 Nov 2004 (fl), *M*. *Serrano et al*. *5482* (NY).

*Capsicum neei* is morphologically most similar to the Bolivian *C*. *minutiflorum* in having stellate yellow corolla and red fruit at maturity. It can be distinguished by the non-geniculate pendent flowering pedicels and the strongly nerved calyx with 10 unequal appendages (Figs 10C, 10E and 11B) versus the geniculate and erect flowering pedicels and the calyx weakly nerved and with 5 equal short appendages in *C*. *minutiflorum* (Rusby) Hunz. The flowers in *C*. *neei* often appear to be solitary but the remains of 2–3 early deciduous bud or flower scars can be seen in the axils. Fruit features as pungency, presence of giant cells and sclerotic granules in the pericarp and mature seeds are unknown at present but it is probable that the fruits are pungent and have giant cells in the innermost layer of the pericarp as occur in the remaining species of the Bolivian clade where *C*. *neei* is positioned.

This new species is sympatric with *C*. *baccatum* L. var. *baccatum*, a taxon with a much wider distribution in South America, that has geniculate pedicels, calyx with 5 equal appendages, white corollas with greenish yellow spots inside and ovoid or globose red fruits.

*Capsicum neei* has been resolved as a new member of the Bolivian clade, which is coherent with its geographic range and the main common feature recognized for the clade, the yellow corollas [1]. However, the Bolivian clade has a weak support, most likely due to the apparent divergence of *C*. *coccineum* from the rest of the species; indeed, *C*. *coccineum* would deserve more attention considering some morphological variability observed in the species (GEB, pers. obs.).

### Final comments on *Capsicum* phylogenetics

The BI tree resolves the same major clades obtained in the previous study of Carrizo García et al. [1]. The position of the three new species included in the current analysis was strongly resolved, therefore just increasing the number of species within the clades where they are placed. However, the affinities of the Bolivian clade, where *C*. *neei* is resolved, need to be further analysed. Indeed, the Flexuosum clade was resolved as its sister group in a previous study [1], in both the BI and MP consensus trees (with strong and weak support, respectively), but that result was not repeated this time. That previous result was already revealing certain weakness on this matter. As regards the Andean clade, where *C*. *piuranum* and *C*. *longifolium* are nested, the latter forms the first branch to diverge within the clade, instead of *C*. *dimorphum*, as previously found [1]. Within this clade, excluding *C*. *longifolium* and *C*. *dimorphum*, two groups of species can be distinguished: *C*. *rhomboideum* and *C*. *hookerianum*, on the one side, and *C*. *lanceolatum*, *C*. *piuranum*, *C*. *lycianthoides* and *C*. *geminifolium*, on the other side (Fig 6). The short length of the branches within the latter group (not counting the *C*. *lanceolatum* branch) would be a signal of the closeness between these species. In fact, the delimitation of/between *C*. *lycianthoides* and *C*. *geminifolium* has required extensive and meticulous herbarium and field observations (GB, pers. obs.). Besides, it is worth mentioning that *C*. *hookerianum* has been included for the first time in a phylogenetic analysis of the genus and, in agreement with earlier hypothesis [1], the species is resolved as a member of the Andean clade. As a final comment, the present results add more evidence about the marked divergence between the Andean clade and the rest of *Capsicum* (Fig 6), already discussed and sustained on chemical, anatomical and cytological features [1], such us non-pungent fruits, without giant cells in the pericarp, and the 2n = 2x = 26 karyotype.

## Supporting information

S1 Table Karyotype features of the *Capsicum* taxa studied (2n = 26). *HKL* haploid karyotype length; χ mean value; *sd* standard deviation (no. of metaphases included in the measurements indicated in S4 Table). Heterochromatin amount expressed as percentage of HKL; *NOR*-*assoc*. NOR-associated heterochromatin; *Interc*. Intercalary heterochromatin.
(DOC)

S2 Table PCR protocols followed for DNA amplification using the Phusion Green Hot Start II High-Fidelity PCR Master Mix. Markers are specified between brackets when conditions differ.
(DOC)

S3 Table Materials and taxa studied in the phylogenetic analysis: Position within *Capsicum* (clade) or as outgroup, provenance, voucher specimens, ID in the BI tree, and GenBank accession numbers for each marker analyzed. Sequences retrieved from GenBank are marked with an asterisk (materials not specified here).
(XLS)

S4 Table Karyotype measurements in C. *longifolium* and C. *piuranum*. Number of seedlings and somatic metaphases analysed per sample, respectively, given in brackets after the voucher number. χ mean value; *sd* standard deviation; *HKL* haploid karyotype length; m metacentric chromosome; *sm* submetacentric chromosome; *st* subtelocentric chromosome; *m-sat* metacentric chromosome with secondary constriction and satellite; *fhcb* fluorochrome heterochromatic band. *p* and *q* upper and lower arms, respectively, in the ideograms (Fig 1). Bands are terminal except those marked with ^, which are intercalary. * Band related to NOR. Figure in brackets below the position of intercalary bands indicates the distance from the centromere in percentage (di).
(DOC)
