# Supplementary material for: Four new species of Capsicum (Solanaceae) from the tropical Andes and an update on the phylogeny of the genus
Source: PLoS One. 2019 Jan 16;14(1):e0209792. doi: 10.1371/journal.pone.0209792 (PMC6334993; doi:10.1371/journal.pone.0209792)
Supplement: S1 Table — HKL haploid karyotype length; χ mean value; sd standard deviation (no. of metaphases included in the measurements indicated in S4 Table). Heterochromatin amount expressed as percentage of HKL; NOR-assoc. NOR-associated heterochromatin; Interc. Intercalary heterochromatin. (DOC) [file pone.0209792.s001.doc]

**S1 Table. Karyotype features of the *Capsicum* taxa studied (2n = 26).**

*HKL* haploid karyotype length; χ mean value; *sd* standard deviation (no. of metaphases included in the measurements indicated in Table 2). Heterochromatin amount expressed as percentage of HKL; *NOR-assoc.* NOR-associated heterochromatin; *Interc.* Intercalary heterochromatin

| Taxon and cytotype | Karyotype formula (n) | Ordering no. of NOR-bearing pairs | HKL (µm)    χ (sd) | Heterochromatin amount Total NOR-assoc. Interc. | Maximum no. of bands per haploid complement | Maximum no. of pairs with bands |
| --- | --- | --- | --- | --- | --- | --- |
| *C. longifolium* | 9 m + 3 sm + 1 st | 10 (sm) | 23.86 (3.77) | 1.48 0.35 0.08 | 18 | 10 |
| *C. piuranum* | 9 m + 3 sm + 1 st | 10 (sm) | 22.97 (2.84) | 1.95 0.45 0.09 | 21 | 11 |
