# Supplementary material for: Four new species of Capsicum (Solanaceae) from the tropical Andes and an update on the phylogeny of the genus
Source: PLoS One. 2019 Jan 16;14(1):e0209792. doi: 10.1371/journal.pone.0209792 (PMC6334993; doi:10.1371/journal.pone.0209792)
Supplement: S2 Table — Markers are specified between brackets when conditions differ. (DOC) [file pone.0209792.s002.doc]

S2 Table. **PCR protocols followed for DNA amplification using the Phusion Green Hot Start II High-Fidelity PCR Master Mix**. Markers are specified between brackets when conditions differ.

| initial denaturation | 35 cycles | | | final extension |
| --- | --- | --- | --- | --- |
|  | denaturation | annealing | extension |  |
| 98°C - 30” | 98°C - 10” | 65°C - 30” (*waxy*)  52°C - 15” (*ndhF*-*rpl32*)  56°C - 20” (*rpl32-trnL*)  61°C - 20” (*psbA-trnH*, *trnL-trnF*) | 72°C - 30” (waxy)  72°C - 20” (*ndhF*-*rpl32, rpl32-trnL*, *psbA-trnH*, *trnL-trnF*) | 72°C - 5’ |
