# Supplementary material for: Four new species of Capsicum (Solanaceae) from the tropical Andes and an update on the phylogeny of the genus
Source: PLoS One. 2019 Jan 16;14(1):e0209792. doi: 10.1371/journal.pone.0209792 (PMC6334993; doi:10.1371/journal.pone.0209792)
Supplement: S4 Table — Number of seedlings and somatic metaphases analysed per sample, respectively, given in brackets after the voucher number. χ mean value; sd standard deviation; HKL haploid karyotype length; m metacentric chromosome; sm submetacentric chromosome; st subtelocentric chromosome; m-sat metacentric chromosome with secondary constriction and satellite; fhcb fluorochrome heterochromatic band. p and q upper and lower arms, respectively, in the ideograms (Fig 1). Bands are terminal except those marked with ^, which are intercalary. * Band related to NOR. Figure in brackets below the position of intercalary bands indicates the distance from the centromere in percentage (di). (DOC) [file pone.0209792.s004.doc]

**S4 Table. Karyotype measurements in *C. longifolium* and *C. piuranum*.**

Number of seedlings and somatic metaphases analysed per sample, respectively, given in parentheses after the voucher number. *χ* mean value; *sd* standard deviation; *HKL* haploid karyotype length; *m* metacentric chromosome; *sm* submetacentric chromosome; *st* subtelocentric chromosome; *m-sat* metacentric chromosome with secondary constriction and satellite; *fhcb* fluorochrome heterochromatic band. *p* and *q* upper and lower arms, respectively, in the idiograms (Fig. 1). Bands are terminal except those marked with ^, which are intercalary. * Band related to NOR. Figure in parentheses below the position of intercalary bands indicates the distance from the centromere in percentage (di).

| Pair | Length | | r  (q/p) | Type | Position of fhcb | Length of fhcb | |
| --- | --- | --- | --- | --- | --- | --- | --- |
|  | Absolute (μm)   X (sd) | Relative (% of HKL) | X (sd) |  |  | Absolute (µm)  X (sd) | Relative (% of HKL) |
| *C. longifolium* GEB & SLG 4821 (2, 5) | | | | | | | |
| 1 | 2.34 (0.38) | 9.82 | 1.21 (0.16) | m | p  q | 0.06 (0.01)  0.06 (0.01) | 0.25  0.25 |
| 2 | 2.12 (0.35) | 8.88 | 1.12 (0.04) | m | p  q | 0.06 (0.01)  0.07 (0.01) | 0.25  0.29 |
| 3 | 2.10 (0.42) | 8.81 | 1.20 (0.07) | m | p  q  q^  (34.78) | 0.06 (0.01)  0.08 (0.01)  0.08 (0.01) | 0.25  0.34  0.34 |
| 4 | 2.04 (0.27) | 8.55 | 1.20 (0.05) | m | p  q | 0.08 (0.01)  0.05 (0.01) | 0.34  0.21 |
| 5 | 2.01 (0.27) | 8.44 | 1.20 (0.11) | m | p | 0.07 (0.02) | 0.29 |
| 6 | 1.84 (0.28) | 7.73 | 1.19 (0.14) | m | p  q | 0.06 (0.01)  0.05 (0.01) | 0.25  0.21 |
| 7 | 1.72 (0.25) | 7.21 | 1.20 (0.12) | m | p  q | 0.06 (0.01)  0.06 (0.01) | 0.25  0.25 |
| 8 | 1.52 (0.33) | 6.39 | 1.02 (0.04) | m |  |  |  |
| 9 | 1.59 (0.24) | 6.68 | 1.56 (0.55) | m | p  q | 0.07 (0.03)  0.05 (0.01) | 0.29  0.21 |
| 10 | 1.86 (0.32) | 7.81 | 1.98 (0.26) | sm-NOR | p* | 0.35 (0.06) | 1.47 |
| 11 | 1.79 (0.45) | 7.50 | 2.66 (0.23) | sm | p | 0.08 (0.02) | 0.34 |
| 12 | 1.56 (0.28) | 6.52 | 2.56 (0.36) | sm |  |  |  |
| 13 | 1.35 (0.30) | 5.66 | 3.08 (0.88) | st |  |  |  |
| *C. piuranum* GEB & SLG 4841 (2, 8) | | | | | | | |
| 1 | 2.02 (0.37) | 8.79 | 1.16 (0.08) | m | q | 0.08 (0.02) | 0.35 |
| 2 | 2.07 (0.36) | 8.99 | 1.20 (0.11) | m | p  q | 0.07 (0.01)  0.08 (0.01) | 0.30  0.35 |
| 3 | 1.93 (0.29) | 8.39 | 1.16 (0.08) | m | p  q  q^  (49.03) | 0.07 (0.02)  0.07 (0.02)  0.09 (0.01) | 0.30  0.30  0.39 |
| 4 | 2.03 (0.33) | 8.84 | 1.14 (0.09) | m | p  q | 0.08 (0.02)  0.07 (0.02) | 0.35  0.30 |
| 5 | 1.88 (0.31) | 8.20 | 1.16 (0.04) | m | p  q | 0.08 (0.02)  0.07 (0.02) | 0.35  0.30 |
| 6 | 1.82 (0.30) | 7.94 | 1.15 (0.08) | m | p  q | 0.07 (0.01)  0.08 (0.02) | 0.30  0.35 |
| 7 | 1.76 (0.26) | 7.67 | 1.26 (0.13) | m | p  q | 0.09 (0.02)  0.07 (0.02) | 0.39  0.30 |
| 8 | 1.71 (0.32) | 7.45 | 1.15 (0.09) | m | p  q | 0.07 (0.02)  0.07 (0.02) | 0.30  0.30 |
| 9 | 1.63 (0.21) | 7.08 | 1.26 (0.38) | m | p  q | 0.09 (0.02)  0.07 (0.02) | 0.39  0.30 |
| 10 | 1.77 (0.25) | 7.71 | 1.72 (0.30) | sm-NOR | p* | 0.45 (0.10) | 1.96 |
| 11 | 1.66 (0.45) | 7.21 | 1.82 (0.12) | sm | p  q | 0.10 (0.03)  0.07 (0.01) | 0.44  0.30 |
| 12 | 1.49 (0.13) | 6.48 | 2.48 (0.40) | sm |  |  |  |
| 13 | 1.21 (0.10) | 5.25 | 3.35 (0.49) | st |  |  |  |
